# A Strategy for the Selection of RT-qPCR Reference Genes Based on Publicly Available Transcriptomic Datasets

**DOI:** 10.3390/biomedicines11041079

**Published:** 2023-04-03

**Authors:** Alice Nevone, Francesca Lattarulo, Monica Russo, Giada Panno, Paolo Milani, Marco Basset, Maria Antonietta Avanzini, Giampaolo Merlini, Giovanni Palladini, Mario Nuvolone

**Affiliations:** 1 Department of Molecular Medicine, University of Pavia, 27100 Pavia, Italy; 2 Amyloidosis Research and Treatment Center, Fondazione IRCCS Policlinico San Matteo, 27100 Pavia, Italy; 3 Pediatric Hematology Oncology, Cell Factory, Fondazione IRCCS Policlinico San Matteo, 27100 Pavia, Italy

**Keywords:** normalization, RT-qPCR, reference gene

## Abstract

In the next-generation sequencing era, RT-qPCR is still widely employed to quantify levels of nucleic acids of interest due to its popularity, versatility, and limited costs. The measurement of transcriptional levels through RT-qPCR critically depends on reference genes used for normalization. Here, we devised a strategy to select appropriate reference genes for a specific clinical/experimental setting based on publicly available transcriptomic datasets and a pipeline for RT-qPCR assay design and validation. As a proof-of-principle, we applied this strategy to identify and validate reference genes for transcriptional studies of bone-marrow plasma cells from patients with AL amyloidosis. We performed a systematic review of published literature to compile a list of 163 candidate reference genes for RT-qPCR experiments employing human samples. Next, we interrogated the Gene Expression Omnibus to assess expression levels of these genes in published transcriptomic studies on bone-marrow plasma cells from patients with different plasma cell dyscrasias and identified the most stably expressed genes as candidate normalizing genes. Experimental validation on bone-marrow plasma cells showed the superiority of candidate reference genes identified through this strategy over commonly employed “housekeeping” genes. The strategy presented here may apply to other clinical and experimental settings for which publicly available transcriptomic datasets are available.

## 1. Introduction

Reverse transcription qPCR (RT-qPCR) is the method of choice for the analysis of expression levels of a limited number of genes of interest thanks to its ease of use, sensitivity, accuracy, and reproducibility [1,2]. Despite the availability of massively parallel sequencing approaches, including RNA sequencing, RT-qPCR remains a widely employed technique for several applications in both basic research and molecular diagnostics [3,4,5,6,7]. Accurate quantification of RNA levels through RT-qPCR critically depends on several variables, including the variability of RNA extraction, variability of reverse transcription, and PCR amplification efficiency [1,8]. Thus, a reliable normalization method that corrects for these variables is essential to ensure accurate quantification through RT-qPCR, and this is most often achieved through the analysis—along with measurements of the genes of interest—of reference genes as internal controls [1,8].

The most commonly employed reference genes, often termed “normalizing genes” or “housekeeping genes”, for normalization of RT-qPCR expression data are *ACTB*, *GAPDH*, *RNA18S*, *B2M,* and *HPRT1* [1,9]. This small set of genes was traditionally employed for normalization purposes in different techniques, such as Northern blotting, RNase protection assays, and conventional reverse transcription PCR, since such genes are thought to be generally stably expressed, based on their biological function [1].

Still, already in the era preceding RT-qPCR or transcriptomic analyses, several reports had described physiological or pathological conditions under which the mRNA levels of these reference genes show significant variation [10,11,12]. Surprisingly, the advent of RT-qPCR, with its potential to accurately quantify gene expression levels, was not accompanied by an assessment of the suitability of these reference genes for the novel purpose, and these classic reference genes were simply adopted in most of the studies as a sort of “historical carryovers” [1]. It is therefore not surprising that numerous RT-qPCR studies have demonstrated that expression of these classic reference genes can substantially vary in different experimental settings and that they are therefore inadequate as reference genes in those cases [13,14,15,16,17,18,19,20,21,22].

Notably, a bioinformatics analysis conducted by Sun and coworkers has demonstrated that human *ACTB* and *GAPDH* and their murine orthologues *Actb* and *Gapdh* display 64, 67, 69, and 197 pseudogenes in the corresponding genomes [23]. More importantly, these pseudogenes are intron-less, similar in size, and highly homologous to the corresponding functional genes, implying that amplification of cDNA from transcribed pseudogenes or gDNA contaminating the cDNA preparation may not be avoided with most primer pairs for these classic reference genes [23]. Furthermore, the choice of inappropriate reference genes may lead to erroneous conclusions [1,17,20,24,25], and has occasionally required publication retractions [26,27,28,29].

The choice of proper reference genes for data normalization is regarded as one of the most critical steps in RT-qPCR experiments [1,2,8,30,31]. The MIQE guidelines (an acronym for Minimum Information for publication of Quantitative real-time PCR Experiments) and experts in the field have endorsed the systematic validation of reference genes in a given experimental setting and the use of multiple, pre-validated reference genes for normalization of RT-qPCR data with proper normalizing algorithms [1,8,30,32]. Nevertheless, these recommendations are not universally adopted [2,8,9,32,33,34].

In the transcriptomic era, the availability of datasets based on microarrays or, more recently, on RNA sequencing experiments provides a means to identify genes with a stable expression in a specific context [35,36,37]. To enable the identification of suitable reference genes based on publicly available and easily accessible transcriptomic datasets, we have exploited the availability of the Gene Expression Omnibus GEO (https://www.ncbi.nlm.nih.gov/geo/, accessed on 26 March 2023), a public repository of functional genomic data, which is part of the National Center for Biotechnology Information (NCBI). Notably, the GEO2R interactive web tool allows users to search for the expression profile of genes of choice within a specific dataset and to obtain sample-level expression data and primary statistics (including adjusted *p*-values, p_adj_). This enables us to obtain a measure of differential expression of a gene across experimental conditions and therefore to identify genes stably expressed in a specific setting.

In this context, we hypothesized that in silico investigation of expression levels of a list of candidate reference genes in a transcriptomic dataset relevant to the case under scrutiny might represent a valuable way to shortlist a set of genes with the most stable expression pattern for subsequent analyses through appropriate RT-qPCR assay design and validation. As a proving ground to test the feasibility of the proposed strategy for the selection of suitable qPCR reference genes, we elected to focus on the study of primary, bone marrow (BM)-derived plasma cells from patients with immunoglobulin light chain (AL) amyloidosis, a rare, life-threatening disorder, caused by a usually small plasma cell (or B cell) clone within the bone marrow [38].

## 2. Materials and Methods

### 2.1. Systematic Review of qPCR Normalizing Genes Used in Human Studies

The published literature on the analysis of candidate reference genes for the normalization of qPCR data in the context of human studies was retrieved through a search in PubMed performed on 1 December 2020. For this purpose, a combination of each of the keywords “Reference genes”, “normalization genes” or “normalization”, with each of the keywords “RT-PCR”, “qPCR”, “real time PCR”, “real time qPCR”, “quantitative PCR” was used. The following PubMed filters were applied: species—humans; search field—title. This search resulted in a list of 144 unique studies.

The abstracts of all the retrieved studies were analyzed and studies not covering the desired topic were excluded (39 studies). The remaining 105 studies matching the target of interest were analyzed to extrapolate the following information: experimental setting, candidate reference genes tested, the normalizing approach used, and best-performing reference gene(s) identified (Supplementary Figure S2).

### 2.2. Interrogation of Gene Expression Omnibus Datasets of Plasma Cell Dyscrasias

The Gene Expression Omnibus (GEO) portal was searched to identify datasets reporting expression data in primary, patient-derived BM plasma cells from patients with AL amyloidosis or other plasma cell dyscrasia compared to healthy control subjects (date of search: 2 February 2021). Two suitable datasets were retrieved. The first dataset (GSE73040) is a phenotypic, transcriptomic, and genomic characterization of clonal plasma cells in light chain amyloidosis, based on a small patient population of AL patients (*n* = 9) and healthy donors (*n* = 5) [39]. The second dataset (GSE6477), based on a large patient population, covered the spectrum of plasma cell dyscrasia, from healthy donors (*n* = 15) to monoclonal gammopathies of undetermined significance (MGUS, *n* = 22), smouldering multiple myeloma (smouldering MM, *n* = 24), newly diagnosed MM (*n* = 73), and relapsed MM (*n* = 28) [40,41].

### 2.3. Primer Design

Primer sequences were designed using NCBI/Primer-BLAST (https://www.ncbi.nlm.nih.gov/tools/primer-blast/, accessed on 26 March 2023), using default parameters for the Refseq mRNA database of Homo sapiens, except the following: PCR product size—min 70 bp, max 120 bp; exon junction span—primer must span an exon–exon junction.

Primer pairs obtained from NCBI/Primer-BLAST were analyzed in Ensembl (https://www.ensembl.org/index.html, accessed on 26 March 2023) and dbSNP (https://www.ncbi.nlm.nih.gov/projects/SNP/, accessed on 26 March 2023) for the presence of known single nucleotide polymorphisms (SNPs) in the corresponding genomic regions. Pairs with a known SNP in the last five positions of the 3′ of one primer were excluded to avoid a negative impact on PCR efficiency [42].

For the same reason, the REDIportal (http://srv00.recas.ba.infn.it/atlas/search.html, accessed on 26 March 2023) was searched to exclude the presence of known A-to-I RNA editing sites within the regions of the transcripts targeted by each primer pair. For this purpose, each target gene was searched in the REDIportal using default parameters, except the following: tissue—all; body site—all. The obtained list of editing sites was inspected to exclude the presence of an editing site in the primer under scrutiny.

Finally, OligoAnalyzer 3.1 website (https://eu.idtdna.com/calc/analyzer, accessed on 26 March 2023) was interrogated using default parameters to check the maximum T_m_ of the most probable hairpin structure made by each primer (T_m_ > 60 °C was not accepted), and the minimum ΔG of homo- and hetero-dimer for each primer and primer pair (ΔG > −10 was not accepted).

This path was iteratively followed until an adequate primer pair was identified for each candidate reference gene. Only if an iteration of this path for 15 consecutive primer pairs obtained through NCBI/Primer-BLAST was unproductive, NCBI/Primer-BLAST was searched with the omission of the filter: exon junction span—primer must span an exon–exon junction, or we tolerated the presence of rare SNP(s) (frequency 1 < 250,000 alleles or unknown frequency). Primer sequences are included in Supplementary Materials.

### 2.4. Cell Culture

The amyloidogenic plasma cell line ALMC-2 [43] was kindly provided by Dr. Diane Jelinek. ALMC-2 cells were cultured in Iscove’s modified Dulbecco medium IMDM 1X+GlutaMAX (Gibco, Life Technologies, Carlsbad, CA, USA), supplemented with 1% pen/strep (penicillin 10,000 units/mL + streptomycin 10,000, Gibco, Life Technologies), 10% heat-inactivated fetal bovine serum (Origin: South America, Gibco, Life Technologies), 1 ng/mL interleukin-6 (IL-6, Gibco, Life Technologies), and 10 ng/mL insulin-like growth factor-I (IGF-I, Gibco, Life Technologies). Cells were seeded at a density of 2–5 × 10^5^ viable cells/mL of medium and grown in a 37 °C incubator with a humidified atmosphere of 5% CO_2_ in the air and split three times/week. Single-molecule real-time sequencing of the M protein (SMaRT M-Seq) [44] was employed to sequence the immunoglobulin heavy and light chain expressed by ALMC-2 cells for cell line authentication purposes. Negativity to *Mycoplasma* spp. contamination was verified using the N-GARDE Mycoplasma Test Kit (Euroclone, Milano, Italy).

### 2.5. Isolation of Primary Bone-Marrow-Derived Plasma Cells

Bone marrow plasma cells were isolated from diagnostic leftovers of bone marrow (BM) aspirates of 9 patients with AL amyloidosis and 3 subjects lacking a BM plasma cell clone. Bone marrow mononuclear cells (MNCs) were purified by density gradient centrifugation, after laying a 1:1 diluted BM sample on Lympholyte-H (Cederlane, Burlington, ON, Canada), washed in FACS buffer (DPBS, 2% FBS, 2.5 mM EDTA). CD138^+^ cells were isolated from MNCs using CD138 MicroBeads, MS columns, and the OctoMACS Separator (all from Miltenyi Biotec, Bergisch Gladbach, Germany). Based on the number of cells obtained in the CD138^+^ fraction, a second round of purification was performed, with the CD138^+^ fraction serving as input for the second MS column. Aliquots of MNCs, CD138^+^**,** and CD138^−^ fractions obtained from the round(s) of purification were used for flow cytometry analysis to assess the starting population of CD138^+^ cells in the BM and to verify the success of the isolation procedure. The rest of the different fractions were pelleted, lysed in 1 mL of TRIzol reagent (Invitrogen, Thermo Fisher Scientific, Waltham, MA, USA), and stored at −80 °C until further processing.

### 2.6. RNA Extraction and cDNA Synthesis

RNAs from ALMC-2 and primary, BM-derived cells were isolated using the RNeasy Mini Kit (Qiagen). Starting from 1 mL of frozen TRIzol samples, 200 µL of chloroform was added per 1 mL of TRIzol used. After the gradient centrifugation, the aqueous phase was transferred to a new tube and one volume of 100% EtOH was added. Then, 700 µL was loaded in an RNeasy Mini spin column and processed according to the manufacturer’s instructions. For all RNAs, concentration and quality were determined with a NanoDrop Spectrophotometer ND1000 (Thermo Fisher Scientific, Waltham, MA, USA) and agarose gel electrophoresis, respectively.

Reverse transcription was performed using the QuantiTect Reverse Transcription kit (Qiagen GmbH, Hilden, Germany), according to the manufacturer’s instructions. To assess the dynamic range of each RT-qPCR assay, 1 μg of total RNA from ALMC-2 cells was retrotranscribed and the resulting cDNA was diluted from 10^−^^1^ to 10^−^^5^. For all other studies (both from ALMC-2 cells and primary, BM-derived cells) 100 ng of total RNA was retrotranscribed and the resulting cDNA was diluted at 10^−^^2^.

### 2.7. Real-Time qPCR

Real-time qPCR was performed on a C1000 Thermal Cycler CFX96 Real-Time system (Biorad, Hercules, CA, USA), using Hard-Shell PCR Plates (96-well, thin-well, white) and Microseal ‘B’ seal (Biorad). Amplification was performed with 7 µL of SsoFast EvaGreen Supermix (Biorad) with 0.5 µM each of forward and reverse primer, 5 µL of cDNA as template, and RNase-free water up to 14 µL of total reaction volume, using the following conditions: 95 °C for 10 min, 40 cycles of 95 °C for 15 s, 60 °C for 1 min, according to the protocol published by Vandesompele and colleagues [45]. Each sample was run in triplicate, except for experiments assessing intra-assay variation, where 6 technical replicates were performed. In each experiment, a no-RT sample and a no-template sample were included as controls.

After each run, a melting curve analysis was performed, using the following conditions: 95 °C for 10 s, and 60 °C to 95 °C for 5 s. For all steps, a ramp rate of 0.5 °C/s was used. In selected experiments, agarose gel electrophoresis was performed to verify the unicity and size of each RT-qPCR amplicon. Details on the adherence to the MIQE guidelines are provided in the Supplementary Materials.

### 2.8. Statistical Analyses

For microarray datasets, adjusted *p*-values generated through the GEO portal were used. All other statistical analyses were performed using GraphPad. The stability ranking of candidate reference genes in primary, BM-derived plasma cells was performed using the RefFinder algorithm [46], a web-based comprehensive tool integrating the currently available major computational programs (geNorm [45], NormFinder [47], BestKeeper [48], and the comparative Δ-Ct method [49]) to compare and rank the tested candidate reference genes (http://blooge.cn/RefFinder/, accessed on 26 March 2023).

## 3. Results

We devised a strategy to select RT-qPCR reference genes based on publicly available and easily accessible transcriptomic datasets and a pipeline to design and validate RT-qPCR assays. This strategy consists of (1) identifying a list of candidate reference genes explored as potential normalizing genes in previous studies, based on a systematic revision of public literature on this topic; (2) ranking candidate reference genes based on their expression profile in previously published transcriptomic datasets closely related to the clinical or experimental setting under study; (3) defining primer pairs for a panel of candidate reference genes with the highest rank based on expression data, following strict requirements in terms of sequence features; (4) performing a technical validation of designed RT-qPCR assays; and (5) selecting the most suited reference genes in the desired clinical or experimental setting using suitable normalizing methods. A schematic representation of the proposed strategy is represented in Supplementary Figure S1.

### 3.1. Identification of Candidate Reference Genes

To compile a list of candidate reference genes for RT-qPCR assays, we performed a systematic revision of published literature focusing on the normalization of RT-qPCR expression data in the context of human studies (Supplementary Figure S2). A total of 163 unique genes were identified from 105 published studies (Supplementary Data). Notably, 5 genes were included in more than 50% of the published studies, with *GAPDH* present in 90% of studies, *ACTB* in 78%, *B2M* in 71%, *HPRT1* in 68%, and *TBP* in 58% (Supplementary Figure S3A). In these publications, the most frequently used normalizing algorithm was geNorm [45], used in 84% of studies, followed by NormFinder [47] in 78%, and BestKeeper [48] in 43% of cases (Supplementary Figure S3B–C).

### 3.2. Filtering of Candidate Reference Genes Based on Plasma Cell Expression Profiles

Next, to identify the most suitable reference genes for RT-qPCR studies on amyloidogenic plasma cells, we assessed the expression profiles of these 163 candidate reference genes in healthy and disease-associated human BM-derived plasma cells based on published transcriptomic studies.

For this purpose, we took advantage of two publicly available global transcriptomic studies of different plasma cell dyscrasias, the GSE73040 [39] and GSE6477 [40,41] datasets. The first dataset (GSE73040) is directly related to the target experimental setting of our study, i.e., amyloidogenic versus control plasma cells. The second dataset (GSE6477) is focused on BM plasma cells with different degrees of malignity and is also relevant for our purposes, considering that AL amyloidosis can occasionally occur in the context of malignant plasma cell clones [50]. Hence, due to the heterogeneity of plasma cell clones in different patients with AL amyloidosis, ideal reference genes for RT-qPCR studies on these cells should display a stable expression pattern over a wide range of plasma cell clonal sizes and proliferation activities.

We then aimed at analyzing the expression profiles of the 163 candidate reference genes in these two datasets. Within each dataset, the candidate reference genes were ranked based on their p_adj_ as a proxy of expression uniformity across the experimental samples, with the highest rank (rank 1) given to the gene with the highest p_adj_ (closest to 1). Nine genes (*CYCA*, *FLJ20030*, *HIST*, *HUPO*, *MT*, *MT-ATP6*, *RPL*, *RPLIA*, and *U6*) had no expression data in both datasets, three genes (*CHCHD1*, *EMC4*, and *TMEM199*) had no expression data only in the GSE6477 dataset, and another gene (*RNA28S1*) had no expression data in the GSE73040 dataset. These genes were excluded from the analysis.

**Figure 1:**
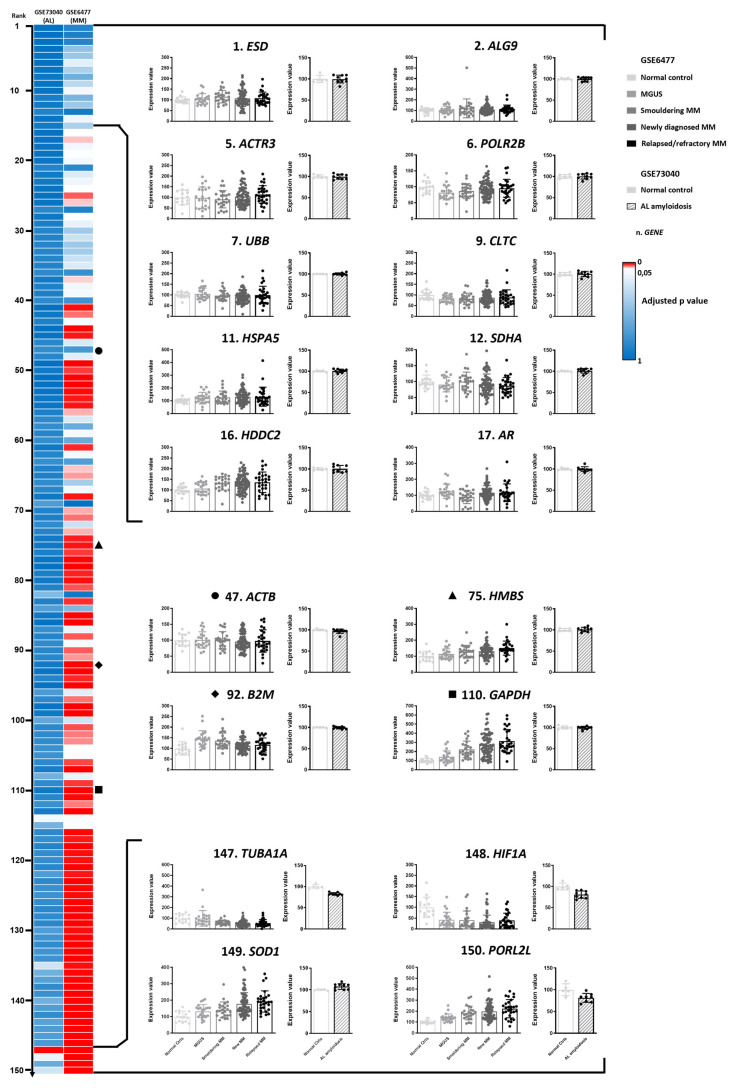
Evaluation of the expression stability of candidate reference genes based on publicly available transcriptomic datasets. Ranking of the candidate reference genes from the systematic research based on their adjusted *p*-values (denoted by the two heatmaps on the left) in the two analyzed gene expression datasets. Of the 163 genes from the systematic search, genes lacking expression data in one or both of the gene expression datasets were excluded. Graphs on the right display the expression levels of genes of selected candidate reference genes with highest ranking positions (upper panel), “classical” reference genes (middle panel, whose relative position in the rank is denoted by four different symbols), or poorly performing genes with the lowest ranking positions (lower panel) in bone-marrow-derived plasma cells from normal controls (Ctrls), patients with monoclonal gammopathy of undetermined significance (MGUS), smoldering multiple myeloma (smoldering MM), newly diagnosed MM (new MM), and relapsed multiple myeloma (relapsed MM) (graphs on the left), or in bone-marrow-derived plasma cells from normal controls (Ctrls) versus patients with AL amyloidosis. The data are derived from GSE6477 (graphs on the left) and GSE73040 (graphs on the right). Within the bar graphs, each dot denotes one subject, the bar denotes the mean, and the error bar denotes the standard deviation. Numbers preceding the gene name (n) denote the composite ranking position based on adjusted *p*-values. Among the candidate reference genes with the highest-ranking positions, genes with a coefficient of variation >45% were excluded (their expression levels in the two datasets are displayed in Supplementary Figure S4).

Of the remaining 150 genes included in this analysis, we noted that 90 genes showed a statistically different expression profile across different diagnostic groups in at least one of these two datasets, more commonly in the GSE6477 dataset (Figure 1). Interestingly, these included *GAPDH*, *B2M*, and *HPRT1*, whose expression was found to be significantly different (adjusted *p*-value, p_adj_ = 1.85 × 10^−12^, 0.00082 and 0.00488, respectively) across the samples of the GSE6477 study. Notably, *GAPDH* was the most differentially expressed gene in this dataset, with a progressive upregulation over the increasing degree of the malignity of the plasma cell clone (Figure 1).

Next, a composite rank was calculated based on the sum of the two ranks in the two datasets. This approach allowed us to focus on the top 18 genes with the best overall performance in the two datasets (Supplementary Table S1). After computing the coefficient of variation of the expression levels of these genes across all study subjects in the two GEO datasets, eight genes (*KDR*, *TUBB*, *CASC3*, *POLR2A*, *BRCA1*, *PF4V1*, *YAP1*, and *YES1*) were found to display a coefficient of variation >45% in one dataset (GSE6477) and were thus excluded from further analyses (Supplementary Figure S4). In parallel, we also investigated five genes among the most commonly used reference genes (*ACTB*, *B2M*, *GAPDH*, *HMBS*, and *HPRT1*, henceforth termed “classical” reference genes) (Supplementary Table S1) for comparison purposes.

### 3.3. Primer Design and Validation

For each gene of interest, we identified a primer pair suitable for RT-qPCR assays. Specifically, we selected primer pairs for the generation of small (70–120 bp) amplicons from human cDNA of the target reference genes having the following characteristics, whenever possible: (1) annealing to exon–exon boundaries or to different exons, thereby reducing the interference of genomic DNA co-purified with RNA and contaminating the cDNA preparations; (2) annealing to regions devoid of single nucleotide polymorphisms or to regions undergoing RNA editing, to avoid reduced qPCR performance due to the presence of single mismatches located ≤ 5 bp from the 3’ end of each primer [42]; (3) no annealing to known pseudogenes with sequence homology to the target gene. Primers and primer pairs were also selected to avoid the formation of thermodynamically relevant hairpins, homo-, or hetero-dimers.

**Figure 2:**
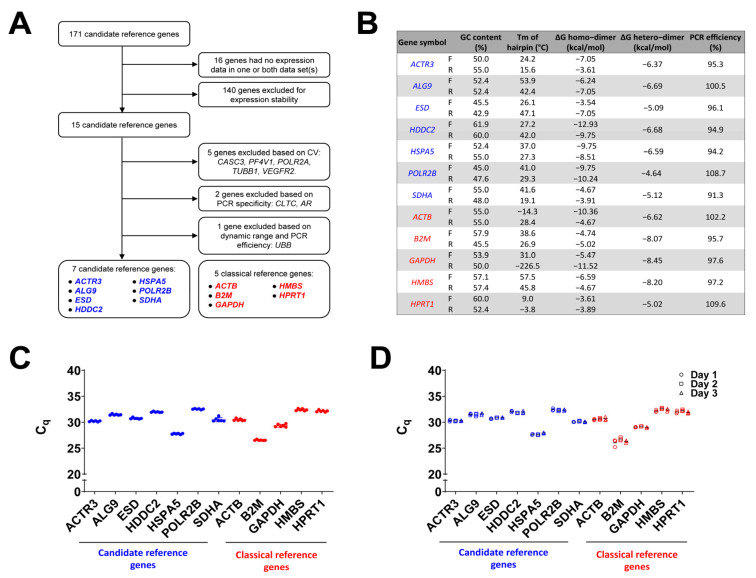
Assay validation for candidate reference genes and the “classical” reference genes. (**A**) The flow chart shows the selection process of candidate reference genes from the initial list of 163 candidates from the systematic search, based on in silico stability analysis (based on adjusted *p*-values and coefficient of variation, CV) and subsequent experimental validation of PCR assays (based on PCR efficiency and dynamic range) until the identification of the seven best-performing candidate reference genes (in blue), whose levels, along with the levels five “classical” reference genes (in red) are explored in AL amyloidosis and control subjects for subsequent analysis. (**B**) The table shows the most relevant parameters impacting on qPCR performance of each of the 12 primer pairs (F: forward primer; R: reverse primer) identified for each of the investigated genes (candidate reference genes in blue; “classical” reference genes in red). (**C**) C_q_ values of six technical replicates, represented by dots, for each candidate reference gene (in blue) or “classical” reference gene (in red) obtained within one single run (intra-assay variation). Horizontal lines denote the mean value for each group. (**D**) C_q_ values of three technical replicates for each candidate reference gene (in blue) or “classical” reference gene (in red) obtained in three different runs on separate days (inter-assay variation). Each symbol (circle, triangle, and square) indicates a different assay/day. Horizontal lines denote the mean value for each group.

PrimerBlast (for primer pair generation and verification of alternative annealing sites), Ensembl (for identification of the annealing region in the gene/transcript context), dbSNP (for identification of known sequence variants in the region), REDIportal (for identification of RNA editing sites), and OligoAnalyzer (for assessment of biophysical properties of primers and primer pairs) were iteratively searched to identify a suitable primer pair for each gene. Overall, 235 primer sequences were analyzed before achieving one primer pair with the desired features for each of the 10 candidate reference genes and the 5 “classical” reference genes (Supplementary Table S2). We used the amyloidogenic plasma cell line ALMC-2 as a source of RNA of human amyloidogenic plasma cells for the technical validation of the selected primer pairs. We used both agarose gel electrophoresis and melting curve analysis to verify PCR specificity for each primer pair and we tested serial dilutions of cDNA over five orders of magnitudes (from 10^−^^1^ to 10^−^^5^) to explore the dynamic range of each assay and calculate PCR efficiency. The PCR specificity was suboptimal for the *AR* gene. The linear dynamic range spanned four orders of magnitude of cDNA dilution for *CASC3* and *RRM1* and five orders of magnitude for the remaining genes. PCR efficiency ranged between 91.3% for *SDHA* and 123.7% for *UBB*. For the subsequent analyses, we focused on the 12 genes (the 7 best-performing candidate genes and the 5 “classical” reference genes) whose PCR assays had a 5-log dynamic range and an efficiency between the ideal interval of 90% and 110% (Figure 2A). No substantial differences were identified between primer pairs of candidate reference genes and of “classical” reference genes in terms of amplicon size, PCR efficiency percentage, GC content percentage, T_m_ of hairpins, and ΔG homo- and hetero-dimers (Figure 2B).

The data on linear dynamic range, PCR efficiency, and specificity are shown in Supplementary Figure S5. For each of the genes under study, we analyzed six technical replicates in one run to assess intra-assay variation. The coefficient for intra-assay variation ranged from 0.27% for *POLR2B* to 1.28% for *SDHA* (Figure 2C). Next, for each gene, we analyzed technical triplicates in three runs on separate days to assess inter-assay variation. The coefficient for inter-assay variation ranged from 0.08% for *ACTR3* to 1.53% for *B2M* (Figure 2D).

### 3.4. Expression Profiles of Investigated Genes in Primary Amyloidogenic and Control Plasma Cells

Next, we collected primary BM-derived plasma cells from diagnostic leftovers of patients with AL amyloidosis with different sizes of the underlying plasma cell clone and different types of the secreted monoclonal protein, as well as from control subjects lacking a BM plasma cell clone.

**Figure 3:**
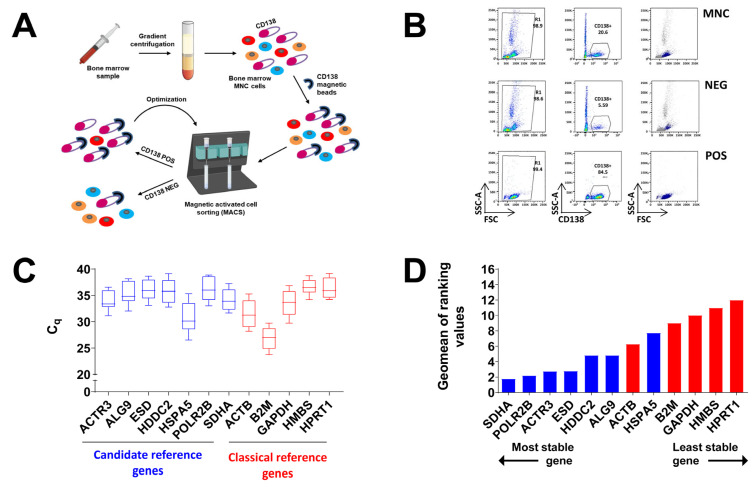
Distribution of expression levels and stability analysis of candidate and “classical” reference genes in AL patients and controls. (**A**) Scheme of immunomagnetic separation of CD138^+^ cells from bone marrow aspirate. MNC: mononuclear cells. CD138 POS: CD138^+^ fraction. CD138 NEG: CD138^-^ fraction. (**B**) Pseudo-color dot plots of flow cytometry analysis of CD138^+^ cells in MNCs as well as negative and positive fractions after the first or second round of purification of a representative patient. (**C**) Distribution of quantification cycle (C_q_) values of candidate reference genes (*n* = 7, in blue) and the “classical” reference genes (*n* = 5, in red) across all the study subjects. Horizontal lines denote median value, lower and upper extremities of boxes denote 25th and 75th percentiles, and lower and upper whiskers denote minimum and maximum values, respectively. (**D**) Stability ranking of candidate reference genes (blue) and “classical” reference genes (red) based on the geomean of ranking values determined by RefFinder across all the study subjects.

For this purpose, we used gradient centrifugation of BM aspirates for MNCs isolation and subsequent magnetic-activated cell sorting with beads coupled to the plasma cell surface marker CD138. One or two sequential rounds of cell sorting were performed in five and seven cases, respectively, based on available cell numbers after the first round of CD138^+^ purification (Figure 3A). The purity of the isolated CD138^+^ fraction was verified by flow cytometry in comparison with the starting population of MNCs and with the CD138^-^ fraction (Figure 3B). In all cases, the percentage of CD138^+^ plasma cells assessed with flow cytometry was ≥70% in the isolated CD138^+^ fraction, except for 1 control subject for which the percentage of CD138^+^ plasma cells in the isolated fraction was 62.5%.

The expression levels of the 12 investigated genes were determined by RT-qPCR in the BM-derived CD138^+^ plasma cells. The tested genes showed a wide range of expression levels across all samples, with mean C_q_ values spanning from 26.93 for gene *B2M* to 37.01 for gene *HPRT1* (Figure 3C). Moreover, the 12 genes displayed distinct expression variability, with *HMBS* showing the narrower range of C_q_ values (minimum C_q_ = 34.2 and maximum C_q_ = 38.7) and *HSPA5* showing the wider one (minimum C_q_ = 26.5 and maximum C_q_ = 35.3) (Figure 3C).

### 3.5. Expression Stability of Investigated Genes in Primary Amyloidogenic and Control Plasma Cells

Lastly, to rank the investigated genes based on the stability of expression across experimental samples, we applied the RefFinder algorithm [46], an analytical tool that runs four of the currently available major computational programs (geNorm [45], NormFinder [47], BestKeeper [48], and the comparative Δ-Ct method [49]), obtains the stability ranking according to each of the four algorithms, and ultimately computes a final stability ranking based on the integration of the four individual rankings. We performed this analysis both for all experimental samples (AL patients and controls) (Figure 3 and Supplementary Figure S7) and for AL patients only (Supplementary Figures S6 and S7). Notably, four of the five “classical” reference genes (*B2M*, *GAPDH*, *HMBS*, and *HPRT1*) systematically occupied the last four positions (9th to 12th, respectively) in the final stability ranking generated by RefFinder, as well as in the four individual rankings generated by geNorm, NormFinder, BestKeeper, and the comparative Δ-Ct method, both when analyzing all experimental samples (AL amyloidosis patients and controls) and when focusing on AL patients only (Figure 3, Supplementary Figures S6 and S7). According to geNorm, all five “classical” reference genes (*ACTB*, *B2M*, *GAPDH*, *HMBS*, and *HPRT1*) occupied the last five positions (8th to 12th, respectively) on the stability ranking, while the ranking position of *ACTB* ranged between 4th and 7th according to the three remaining algorithms and was 7th in the final ranking established by RefFinder (Figure 3, Supplementary Figures S6 and S7). Conversely, the candidate reference genes identified through our strategy typically occupied the first positions within the stability ranking, with the relative ranking positions changing slightly according to the combination of the analytical tool and experimental dataset employed (Figure 3, Supplementary Figures S6 and S7). Overall, these data show that genes identified through our developed strategy typically outperform commonly used normalizing genes for RT-qPCR normalization purposes, demonstrating the utility of our approach.

## 4. Discussion

In the present study, we devised a strategy to select RT-qPCR reference genes based on publicly available and easily accessible transcriptomic datasets and a pipeline to design and validate RT-qPCR assays.

The first step of our strategy consisted of a systematic revision of published literature to identify genes previously investigated as potential reference genes in RT-qPCR studies. The obtained list of candidate reference genes reflects our search criteria, and it is possibly non-exhaustive of all the genes investigated in the literature for this purpose. This list can be easily expanded with other genes, including genes indicated as potential universal reference genes based on the analysis of large transcriptomic datasets [51,52]. Moreover, we have restricted our analysis to studies performed on human cells and tissues. However, it has been proposed that genes stably expressed in a specific cell type or tissue of one species are likely to show the same stable pattern of expression in the same cell type or tissue in other species (a concept termed the ontology clause [52,53]). Hence, based on this assumption, our list could be expanded with reference genes tested in other species and, *vice versa*, it could be applied to studies on other species.

A striking, yet not unexpected, result of our systematic revision is that a small set of candidate reference genes, namely *GAPDH, ACTB, B2M, HPRT1,* and *TBP*, appear in the majority of studies. This is in line with previous literature surveys and confirms the popularity of these as normalizing genes in RT-qPCR studies [1,9,34]. Hence, this popularity persists despite the accruing evidence from the published literature that some of these genes often display variable expression in different experimental settings [13,14,15,16,17,18,19,20,21,22].

Accordingly, 90 out of the 163 candidate reference genes showed a statistically different expression across experimental samples in at least one of the two datasets. This observation implies that researchers arbitrarily selecting a single reference gene for normalization of RT-qPCR experiments with primary BM-derived plasma cells without pre-validation of the chosen gene are at risk of using a reference gene whose expression is differentially modulated within the experimental setting. This is even more worrisome when considering that *GAPDH, B2M,* and *HPRT1*—that is, three of the most widely employed reference genes for RT-qPCR studies—are significantly modulated across different stages of plasma cell malignity. Overexpression of *GAPDH* in this setting is in line with the observation of the upregulation of this gene in proliferating cells and aggressive cancers [13,54].

It should be noted that our list of candidate reference genes comprises several genes that were tested in only one or a few studies as possible reference genes. This implies that our list of candidate reference genes may comprise genes for which the evidence of stable expression across different conditions may be weak. While the inclusion of these genes with limited literature as RT-qPCR reference genes could be regarded as a limitation, it should be noted that our proposed strategy is quite insensitive to the inclusion of poorly performing reference genes in the initial list of genes to be tested. Indeed, the subsequent analysis and ranking of listed candidate reference genes based on related transcriptomic datasets should unveil poorly performing genes with unstable patterns in the investigated clinical or experimental setting.

One important feature of our proposed strategy is the use of published transcriptomic datasets, which are easily accessible through the GEO portal. As explained in the portal (https://www.ncbi.nlm.nih.gov/geo/info/geo2r.html, accessed on 26 March 2023), GEO2R does not rely on curated datasets and directly interrogates the original data file. While this enables a higher proportion of GEO data to be rapidly analyzed, it is important to understand that this research tool can access and analyze almost any GEO series, regardless of data type and quality.

In 2011, Hruz et al. established RefGenes, a platform enabling the selection of suitable reference genes for RT-qPCR studies based on microarray datasets. This tool is accessible only to registered users with a basic (free for academics) or professional license. This, and other microarray-based resources, are currently limited by the rapid rise of RNA sequencing and the decline in microarrays for transcriptomic studies [35].

Conversely, GEO is a public functional genomics data repository maintained by the NCBI and includes both microarray- and RNA-sequencing-based transcriptomic datasets. Therefore, compared with other privately maintained repositories of transcriptomic datasets, especially those restricted to microarray data, GEO ensures unrestricted public access to growing numbers of both microarray and RNA sequencing datasets. Several studies have exploited GEO to interrogate microarrays or, more recently, RNA sequencing datasets to identify genes with stable expression in a specific context [36,37].

Transcriptomics datasets available through GEO can also be mined in parallel through ScanGEO, thus facilitating and expediting the analysis of multiple genes of interest across different studies, and also for the identification of candidate reference genes [22,55]. Nevertheless, our proposed strategy may be adapted to the interrogation of repositories of transcriptomic datasets other than those available through GEO.

Once we had shortlisted the candidate reference genes deserving further investigations in our experimental setting in silico, we needed to investigate their expression in our samples.

Besides the normalization approach, another main determinant of accurate quantification of gene expression through RT-qPCR is the performance of employed RT-qPCR assays, for both the gene(s) of interest and the normalizing gene(s). This crucially depends, among other aspects, on primer design [31]. While some aspects of primer design for RT-qPCR assays are well known and implemented in most RT-qPCR studies, including the need to design primers in exon–exon spanning regions or in different exons separated by large introns, whenever possible, to avoid amplification of genomic DNA co-purified with RNA, other aspects are less known and often disregarded. This is, for example, the case with the potential effect of single base substitutions in the target region against which a primer has been designed. Indeed, recent work had clearly shown that SNPs and other mismatches can reduce the performance of qPCR assays [42]. Hence, when designing novel primer pairs using PrimerBlast, we elected to verify with Ensembl/dbSNP the absence of known SNPs in the corresponding genomic regions where the 3′ region of each primer (namely the last five bases from the 3′ terminus) is predicted to anneal. This requirement was intended to avoid a misalignment of the primer to the cDNA that would interfere with PCR amplification, thereby leading to the underestimation of expression levels in samples from individuals carrying a certain SNP [42]. This task proved to be particularly challenging, in light of the high number and granular distribution of known SNPs in the different candidate reference genes examined (an average of 1 SNP per 58 bp in the whole genome, or 1 SNP per 20 bp in the exome, based on dbSNP release 137 [42]). This clearly reflects the recent flourishing of whole-genome or whole-exome sequencing studies, which are dramatically increasing the knowledge of sequence variation in humans. Indeed, to design 15 primer pairs fulfilling our quality criteria for each of the 10 candidate reference genes to be investigated and the five “classical” reference genes, we had to screen 235 primer pairs, with the presence of known SNPs in the last 5 bases of a primer being the reason for exclusion of a primer pair in almost all cases.

Moreover, RNA editing is another possible source of sequence variation in transcripts which could potentially lead to primer misalignment and impact PCR amplification. RNA editing is a post-transcriptional modification, which alters the RNA sequence, most commonly by adenosine deamination resulting in inosine, which is processed equivalently to guanosine [56,57]. Hence, we verified that none of our designed primers would bind to a predicted RNA editing site, taking advantage of REDIportal, a comprehensive database of A-to-I RNA editing events in humans [58]. Only exceptionally were primer pairs excluded because they were not meeting the biophysical requirements.

As proof-of-principle for the validity of the proposed approach, we applied this strategy for the identification and validation of RT-qPCR reference genes in studies on primary, BM-derived plasma cells of patients with AL amyloidosis. This is a particularly challenging setting from both a biological and an analytical point of view.

In biological terms, the underlying plasma cell clone can significantly vary in terms of size, proliferation activity, and biological malignity [50]. It should be noted that in about 5–7% of cases, AL amyloidosis can occur in the context of IgM-secreting clones (hence more immature, CD138^-^ elements), occasionally in association with Waldenström’s macroglobulinemia [59], as well as in association with other hematologic disorders (e.g., non-Hodgkin lymphoma, chronic lymphocytic leukemia) in exceptional cases [60]. In the present study, we have focused on the analysis of amyloidogenic plasma cell clones, secreting non-IgM monoclonal proteins, which represent 93–95% of cases of AL amyloidosis. In light of the biological difference between IgM and non-IgM clones, it is anticipated that IgM-secreting amyloidogenic clones would require ad hoc investigations for the identification of stably expressed reference genes.

In analytical terms, our chosen experimental setting represents several levels of complexity. First of all, bone marrow as a starting material displays a higher degree of variation compared to other tissues (e.g., peripheral blood). This is not only for the possible patchy distribution of clonal plasma cells in different bone districts but also in relation to aspects of bone marrow sampling (for example, variable degrees of contamination with peripheral blood). Moreover, the isolation of our target cell type, CD138^+^ bone marrow plasma cells, requires a relatively long and complex protocol, including density centrifugation, labeling with immunomagnetic beads, and one or two rounds of column separation. Moreover, the purity of isolated fraction is typically <100% and can vary from case to case. Hence, preclinical variation is expected to be considerably high compared to other clinical or experimental settings. Another important aspect is the small size of the plasma cell clone, as well as the small size of the starting material for isolation (that is, diagnostic leftover from a standard bone marrow aspirate), imposing the need to work with limited amounts of cDNA. In addition, aneuploidy due to the presence of numerical chromosomal alterations is frequently seen in amyloidogenic plasma cells [61,62,63,64,65,66]. Cytogenetic aberrations can result in dysregulated expression of genes located in the affected chromosomes (see for example [67]). These observations justify the use of multiple, pre-validated reference genes, to avoid the consequences of the abnormal expression (or even lack of expression, in the case of loss of genomic material) of an individual reference gene caused directly or indirectly by a cytogenetic abnormality in a patient’s plasma cell clone.

Taking into consideration all these aspects, our strategy enabled us to identify candidate reference genes with superior stability with respect to “classical” reference genes as assessed by multiple, independent algorithms evaluating gene expression stability. Further studies would be needed to verify if these genes and related qPCR assays might be useful for qPCR normalization purposes in other clinical or experimental settings [52,53].

## 5. Conclusions

In conclusion, we have developed a strategy for selecting appropriate reference genes for the normalization of RT-qPCR experiments based on publicly available and easily accessible transcriptomic datasets and a pipeline to design and validate RT-qPCR assays. As proof-of-principle, we have applied this strategy to identify stable reference genes for the analysis of primary, patient-derived amyloidogenic, and control plasma cells, showing that genes identified through this strategy typically outperform commonly used normalizing genes for gene normalization purposes.

## Data Availability

Published transcriptomic datasets analyzed in this study (GSE73040 [39] and GSE6477 [40,41]) can be accessed through the Gene Expression Omnibus GEO portal (https://www.ncbi.nlm.nih.gov/geo/, accessed on 26 March 2023). Data generated within this study are included in the manuscript and Supplementary Materials. Studies obtained through the systematic review of published literature and analyzed within this study are quoted in the References section [16,22,45,47,68,69,70,71,72,73,74,75,76,77,78,79,80,81,82,83,84,85,86,87,88,89,90,91,92,93,94,95,96,97,98,99,100,101,102,103,104,105,106,107,108,109,110,111,112,113,114,115,116,117,118,119,120,121,122,123,124,125,126,127,128,129,130,131,132,133,134,135,136,137,138,139,140,141,142,143,144,145,146,147,148,149,150,151,152,153,154,155,156,157,158,159,160,161,162,163,164,165,166,167,168]. A complete list of these studies is also available as Supplementary Data.

## Supplementary Materials

The following supporting information can be downloaded at: https://www.mdpi.com/article/10.3390/biomedicines11041079/s1: Supplementary Figure S1. Strategy for the identification of RT-qPCR reference genes based on published transcriptomic datasets; Supplementary Figure S2. Workflow of the systematic review; Supplementary Figure S3. Systematic review of the practices of normalization of RT-qPCR experiments; Supplementary Figure S4. Candidate reference genes excluded based on the coefficient of variation analysis; Supplementary Figure S5. Dynamic range, PCR efficiency, and specificity of candidate reference genes and “classical” reference genes; Supplementary Figure S6. Distribution of expression levels and stability analysis of candidate and “classical” reference genes in AL amyloidosis patients; Supplementary Figure S7. Stability analysis of candidate and reference genes according to the comparative Δ-Ct method, BestKeeper, geNorm, and NormFinder; Supplementary Table S1: Features of investigated genes.; Supplementary Table S2. Primer sequences. Supplementary Table S3: MIQE guideline checklist.
