# Supplementary material for: A Strategy for the Selection of RT-qPCR Reference Genes Based on Publicly Available Transcriptomic Datasets
Source: Biomedicines. 2023 Apr 3;11(4):1079. doi: 10.3390/biomedicines11041079 (PMC10135859; doi:10.3390/biomedicines11041079)
Supplement: Supplementary file 1 [file biomedicines-11-01079-s001.zip › biomedicines-2255796-supplementary.pdf]

A strategy for the selection of RT-qPCR reference genes based on publicly available transcriptomic data sets

Alice Nevone [1,2], Francesca Lattarulo [1,2], Monica Russo [1,2], Giada Panno [1,2], Paolo Milani [1,2], Marco Basset [1,2], Maria Antonietta Avanzini [3], Giampaolo Merlini [1,2], Giovanni Palladini [1,2] and Mario Nuvolone [1,2*]

[1] Department of Molecular Medicine, University of Pavia, Pavia, Italy

[2] Amyloidosis Research and Treatment Center, Fondazione IRCCS Policlinico San Matteo, Pavia, Italy

[3] Pediatric Hematology Oncology, Cell Factory, Fondazione IRCCS Policlinico San Matteo, Pavia, Italy

Supplementary Materials

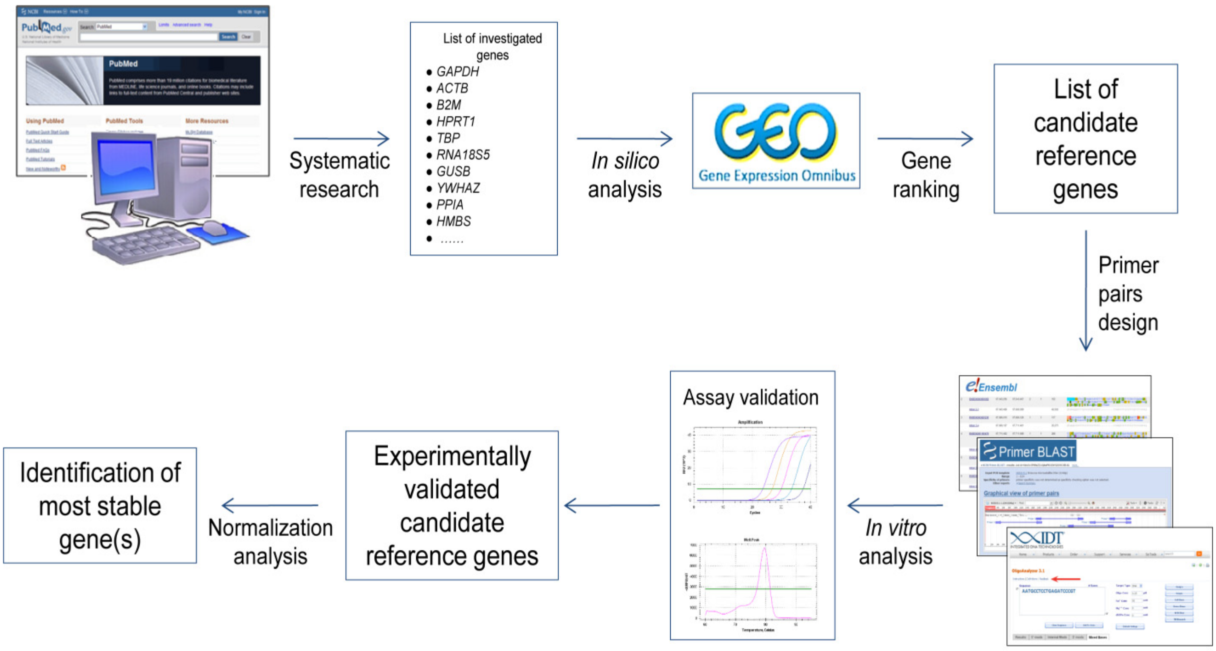

Supplementary Figure S1. Strategy for the identification of RT-qPCR reference genes based on published transcriptomic datasets.

Scheme of the proposed strategy for the identification of appropriate reference genes for RT-qPCR studies based on published transcriptomic datasets.

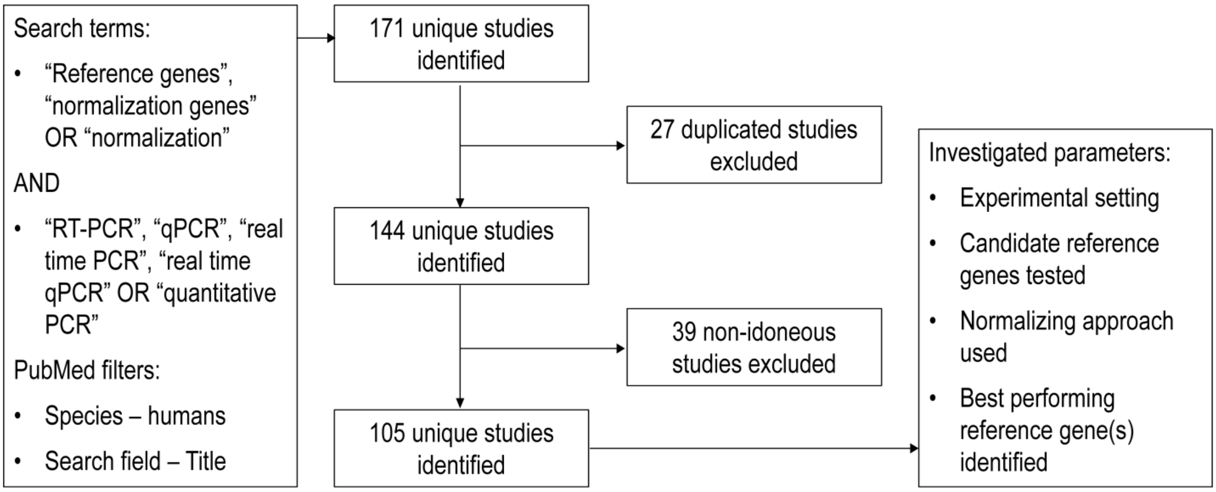

Supplementary Figure S2. Workflow of systematic review

Workflow of the systematic review of published literature on the analysis of candidate reference genes for normalization of qPCR data in the context of human studies.

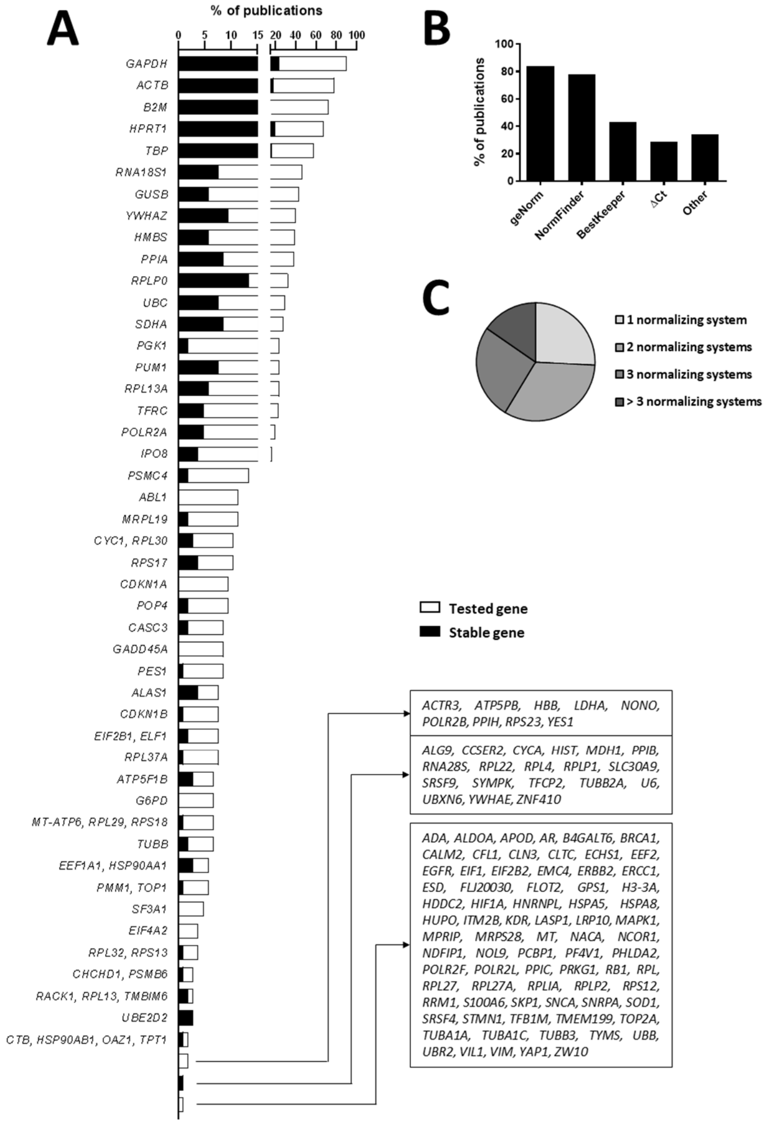

Supplementary Figure S3. Systematic review on practices of normalization of RT-qPCR experiments

A) For each gene or group of genes, the white bar shows the frequency of studies in which the given gene(s) was (were) tested, while the black bar shows the frequency of studies in which the tested gene(s) of interest ranked among the most stable ones. **B**) Frequency of usage of normalizing systems. The sum is higher than 100% as several normalizing systems are used in one publication; **C**) Pie chart shows the number of normalizing system(s) employed in each

publication. **A-C** are referred to the 105 studies retrieved through the systematic literature review.

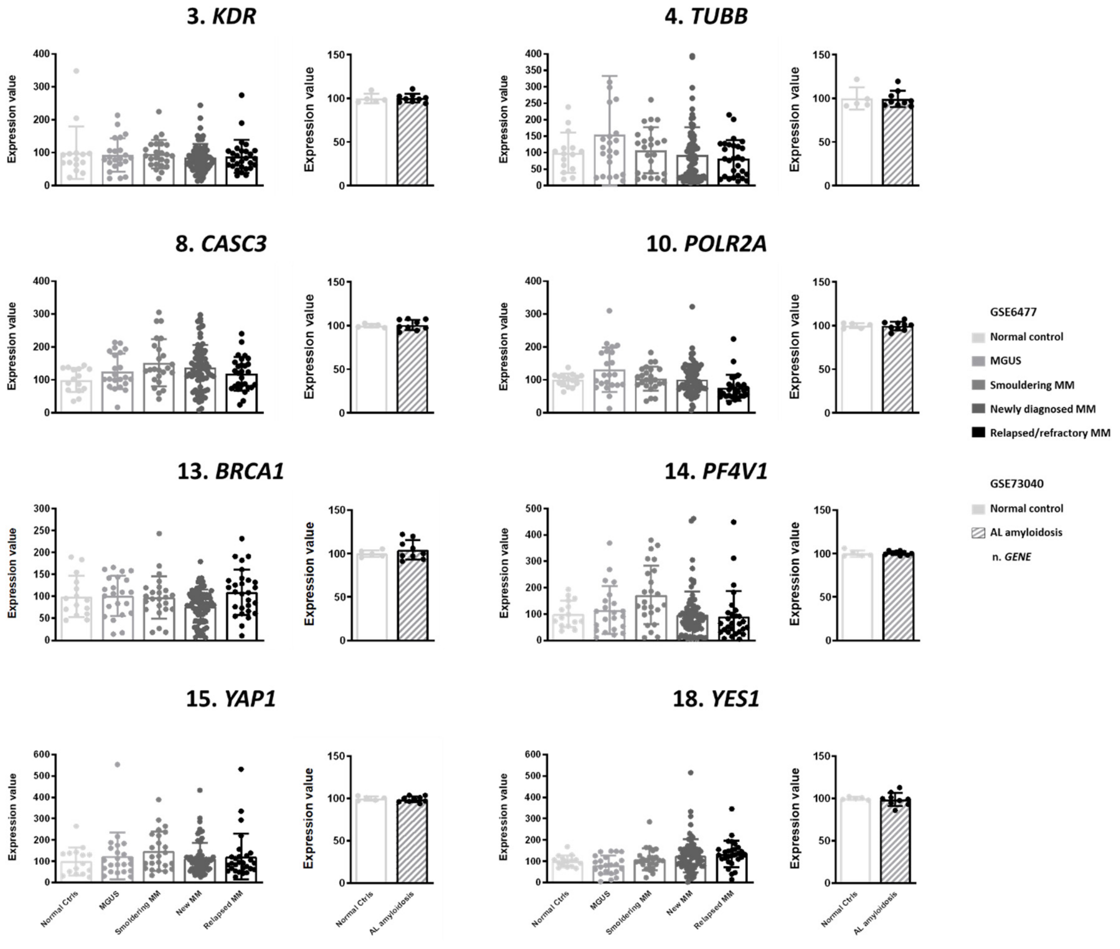

Supplementary Figure S4. Candidate reference genes excluded based on the coefficient of variation analysis.

Expression levels of the candidate reference genes excluded based on the coefficient of variation analysis, across different stages of plasma cells malignity, in bone marrow-derived plasma cells from normal controls (Ctrls), patients with monoclonal gammopathy of undetermined significance (MGUS), smouldering multiple myeloma (smouldering MM), newly diagnosed multiple myeloma (new MM) and relapsed multiple myeloma (relapsed MM) (graphs on the left) or in bone marrow-derived plasma cells from normal controls (Ctrls) versus patients with AL

amyloidosis. Data are derived from GSE6477 (graphs on the left) and GSE73040 (graphs on the right). Each dot denotes one subject, the bar denotes the mean and the error bar denotes the standard deviation. Numbers preceding the gene name denote the composite ranking position based on adjusted *p*-values, as reported in Figure 1.

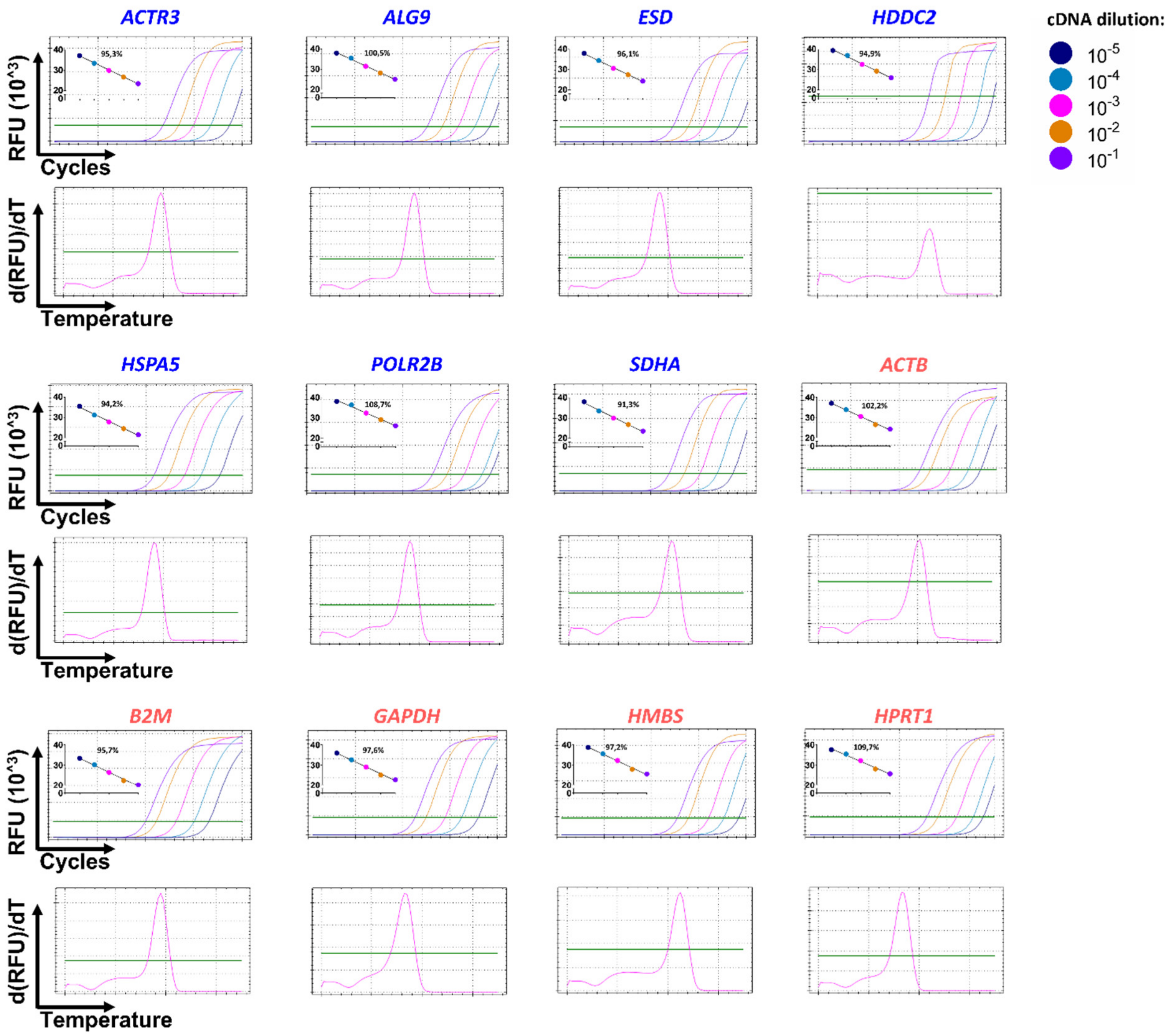

Supplementary Figure S5. Dynamic range, PCR efficiency, and specificity of candidate reference genes and "classical" reference genes.

For each candidate reference gene (blue) and the "classical" reference gene (red), the upper plot shows the relative fluorescence unit (RFU 10^3) over cycle for the different cDNA dilutions (color-coded as in the legend), and in the left corner a plot of the correlation of cDNA dilution and C_q indicates PCR efficiency; the lower plot shows the first negative derivative of RFU (d(RFU)/dT) over the different temperature of the melting curve analysis. Dynamic range: RFU is equal to 16

for *HDDC2*, 20 for *ACTB*, 25 for *GAPDH*, *HMBS,* and *HPRT1*, 30 for *B2M*, 40 for *ACTR3*, *ALG9*, *ESD*, *POLR2B*, *SDHA*, 50 for *HSPA5*; melting curve: -d(RFU)/dT *ALG9*, *ESD*, *POLR2B* (8000), *ACTR3* (7000), *SDHA* (6000), *B2M* and *HPRT1* (5000), *GAPDH* and *HMBS* (4000), *ACTB* (3000), *HDDC2* (2500) and *HSPA5* (10).

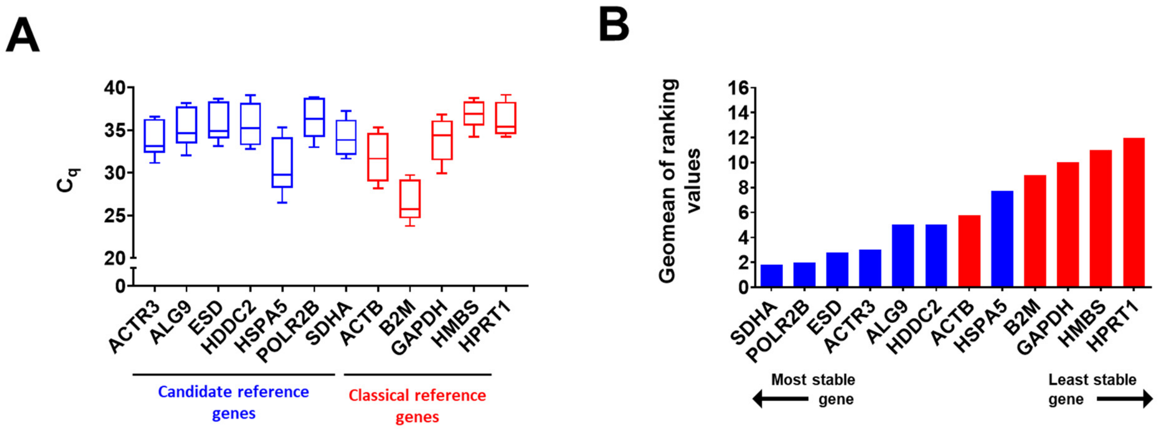

Supplementary Figure S6. Distribution of expression levels and stability analysis of candidate and "classical" reference genes in AL amyloidosis patients.

A) Distribution of quantification cycle (C_q) values of candidate reference genes (*n*=6, in blue) and the "classical" reference genes (*n*=5, in red) in AL amyloidosis patients. Horizontal lines denote median value, lower and upper extremities of boxes denote 25th and 75th percentiles and lower and upper whiskers denote minimum and maximum values, respectively; **B**) Stability ranking of candidate reference genes (blue) and "classical" reference genes (red) based on the geomean of ranking values determined by RefFinder in AL amyloidosis patients.

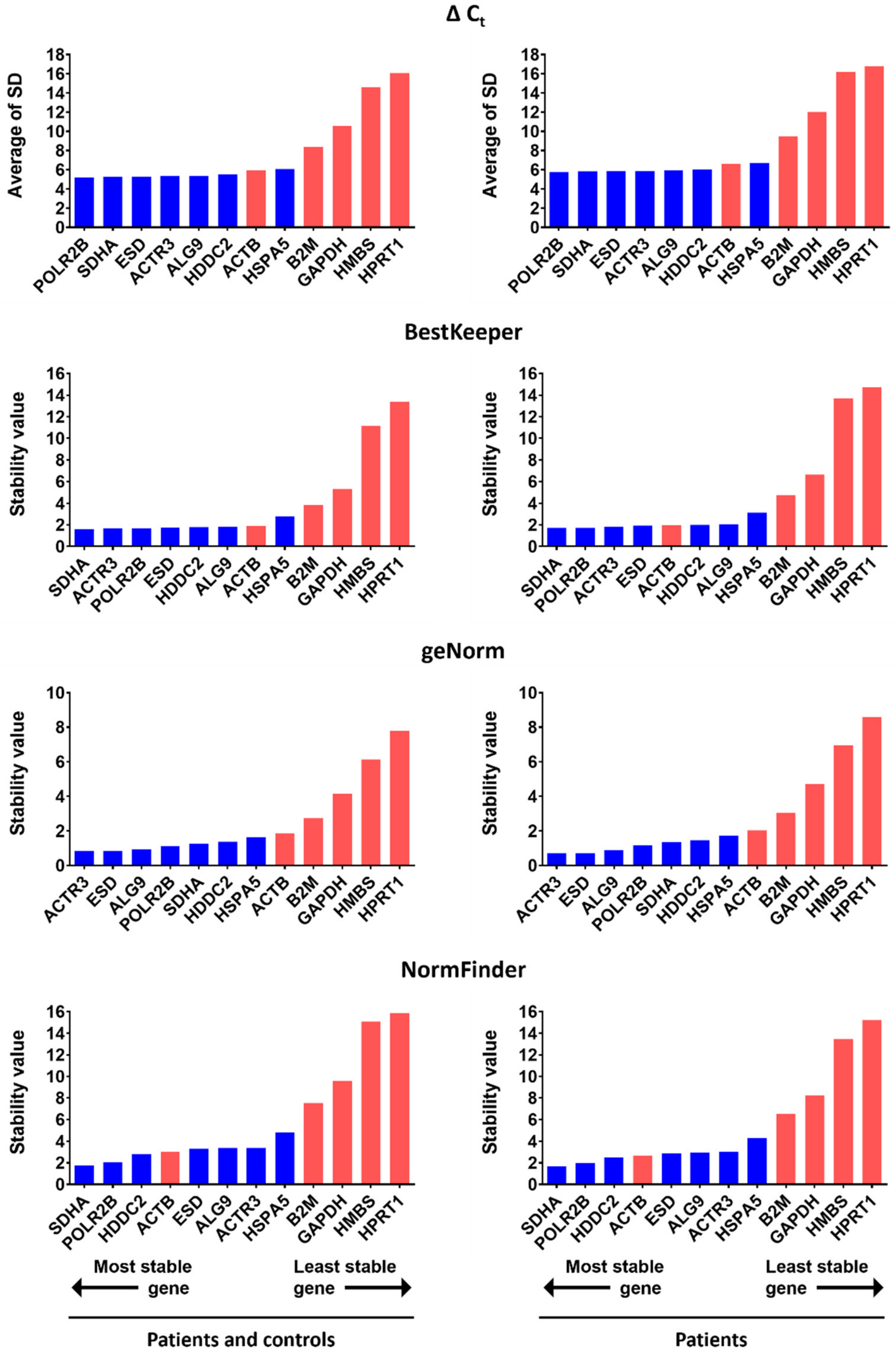

Supplementary Figure S7. Stability analysis of candidate reference genes and "classical" reference genes according to the comparative Δ-C_t method, BestKeeper, geNorm, and NormFinder.

Stability ranking of candidate reference genes (blue) and “classical” reference genes (red) according to the comparative Δ-C_t method, BestKeeper, geNorm, and NormFinder, when analyzing AL amyloidosis patients and controls (Patients and controls, left panels) and when focusing on AL patients only (Patients, right panels).

Supplementary Table S1. Features of investigated genes.

Gene symbol	Accession number	Gene name	Alias	Function	Location	Pseudogene
ACTR3	NM_005721.4	Actin related protein 3	*ARP3*	Major constituent of the ARP2/3 complex	2q14.1	Yes
ALG9	NM_024740.2	α-1,2-mannosyltransferase	*CDG1L, DIBD1, GIKANIS, LOH11CR1J*	Lipid-linked oligosaccharide assembly	11q23.1	No
AR	NM_000044.4	Androgen receptor		Steroid-hormone activated transcription factor	Xq12	No
BRCA1	NM_001407571.1	BRCA1 DNA repair associated	*IRIS, PSCP, BRCAI, BRCC1, FANCS, PNCA4, RNF53, BROVCA1, PPP1R53*	Nuclear phosphoprotein involved in maintaining genomic stability and acts also as a tumor suppressor	17q21.31	
CASC3	NM_007359.4	Cancer susceptibility 3	*MLN51*	Core component of the exon junction complex (EJC)	17q21.1	No
CLTC	NM_004859.3	Clathrin, heavy polypeptide (Hc)	*Hc, CHC, CHC17, MRD56, CLH-17, CLTCL2*	Major protein of the polyhedral coat of coated pits and vesicles	17q23.2	No
ESD	NM_001984.1	Esterase D	*FGH*	Serine hydrolase of the esterase D family	13q14.2	Yes
HDDC2	NM_016063.2	HD domain containing 2		Unknown function	6q22.31	No

HSPA5	NM_005347.4	Heat shock protein family A (Hsp70) member 5	*BIP, GRP78, HEL-S-89n*	Chaperone protein for protein folding and assembly in the ER	9q33.3	Yes
KDR	NM_002253.2	Vascular endothelial growth factor receptor	*VEGFR2*	Cell-surface receptor for VEGFA, VEGFC, and VEGFD	4q12	No
PF4V1	NM_002620.4	Platelet factor 4 variant 1	*PF4A, CXCL4L1, CXCL4V1, PF4-ALT, SCYB4V1*	Chemokine with strong antiangiogenic function	4q13.3	Yes
POLR2A	NM_000937.5	RNA polymerase II subunit A	*RPB1, RPO2, POLR2, POLRA, RPBh1, RPOL2, NEDHIB, RpIILS, hsRPB1, hRPB220*	Largest subunit of RNA polymerase II, responsible for synthesizing messenger RNA in eukaryotes.	17p13.1	No
POLR2B	NM_000938.2	RNA polymerase II subunit B	*RPB2, POL2RB, hRPB140*	Second largest subunit of the DNA-dependent RNA polymerase II (Pol II)	4q12	No
SDHA	NM_004168.3	Succinate dehydrogenase complex flavoprotein subunit A	*FP, PGL5, SDH1, CMD1GG, SDH2, SDHF, MC2DN1, NDAXOA*	Catalytic subunit of succinate-ubiquinone oxidoreductase	5p15	Yes
TUBB	NM_030773.3	Tubulin beta 1 class VI	*M40, TUBB1, TUBB5, CDCBM6, CSCSC1*	Component of microtubules	20q13.32	Yes
UBB	NM_018955.3	Ubiquitin B		Involved in protein degradation	17p11.2	Yes
YAP1	NM_001130145.3	Yes1 associated transcriptional regulator	*YAP, YKI, COB1, YAP2, YAP-1, YAP65*	Downstream nuclear effector of the Hippo signaling pathway	11q22.1	No
YES1	NM_005433.4	YES proto-oncogene 1, Src family tyrosine kinase	*Yes, c-yes, HsT441, P61-YES*	Protein with tyrosine kinase activity, that	18p11.32	No

				belongs to the src family of proteins		
ACTB	NM_001101.5	Actin Beta	*BRWS1, PS1TP5BP1*	Involved in cell motility, structure, integrity, and intercellular signaling	7p22.1	Yes
B2M	NM_004048.4	Beta-2-microglobulin	*IMD43*	Serum protein found in association with the MHC class I heavy chain.	15q21.1	No
GAPDH	NM_001289746.2	Glyceraldehyde-3-phosphate dehydrogenase	*G3PD, GAPD, HEL-S-162eP*	Member of the glyceraldehyde-3-phosphate dehydrogenase protein family	12p13.31	Yes
HMBS	NM_001258209.2	Hydroxymethylbilane synthase	*UPS, PBGD, PORC, PBG-D*	Member of the hydroxymethylbilane synthase superfamily	11q23.3	No
HPRT1	NM_000194.3	Hypoxanthine phosphoribosyltransferase 1	*HPRT, HGPRT*	Catalyze the transfer of 5-phosphoribosyl group from 5-phosphoribosyl 1-pyrophosphate	Xq26.2-q26.3	Yes

Data regarding the 18 candidate reference genes (blues) and 5 “classical” reference genes (red) were retrieved from Ensembl (Pseudogene) and NCBI Gene (all other columns).

Supplementary Table S2. Primer sequences.

Gene symbol	Forward primer (5' -> 3')	Reverse primer (5' -> 3')	Amplicon size (bp)	Exon-exon junction spanning
ACTR3	ACACACCACATGCAGCGATA	TTGGTAGAACTCAGGCGTGG	72	Yes
ALG9	TCATCCAGCCTCACAAAGAGG	CTGTGCTGAAGTGCAGAGAGA	94	Yes
AR	ACTCTGGCTTCACAGTTTGGA	CAGGTCTTCTGGGGTGGAAA	78	Yes
CLTC	AGCAAAGTAATTGCACTGAAAGGTA	AAAGGTGACATCATCAGTCATGG	114	No
ESD	TGTCTACTTACCACCAAAGGCA	TTGCTCTGTGCAAGTTAAACC	79	Yes
HDDC2	CGGTCCCTACTGCAGTTCCTG	CTCTCCGGCCTCTGGACATT	92	No
HSPA5	CAACATGGATCTGTTCCGGTC	TGGAATTCGAGTCGAGCCAC	118	Yes
POLR2B	TGAAATCACCCCGGATTTGT	TCCAGCTGTTGTCTAACCAAG	90	No
SDHA	GAAGCCCTTTGAGGAGCACT	GTCTATATTCCAGAGTGACCTTCCC	83	Yes
UBB	TTGTGTCCCTGTGGGTGGAC	CTGCATTTTGACCTGTTAGCGG	71	Yes
ACTB	CGCGAGAAGATGACCCAGAT	AGGGATAGCACAGCCTGGAT	80	Yes
B2M	GGCTATCCAGCGTACTCCA	AATGTCGGATGGATGAAACCCA	109	Yes
GAPDH	CATTGACCTCAACTACATGGTTTAC	CTTGACGGTGCCATGGAATTTG	73	Yes
HMBS	TTCCTGAGGCACCTGGAAGGA	AGACTCCTCCAGTCAGGTACAG	91	Yes
HPRT1	CCCTGGCGTCGTGATTAGTG	CACCCTTTCCAAATCCTCAGC	91	Yes

Primer sequences of the 10 candidate reference genes (blue) and 5 "classical" reference genes (red). Underlined letters denote bases annealing with a genomic region with a known SNP, according to Ensembl and dbSNP.

Supplementary Table S3. MIQE guideline checklist.

ITEM TO CHECK	IMPORTANCE	CHECKLIST
**EXPERIMENTAL DESIGN**		
Definition of experimental and control groups	E	AL amyloidosis patients and control subjects
Number within each group	E	9 patients with AL amyloidosis and 3 control subjects
Assay carried out by core lab or investigator's lab?	D	Investigator's lab
Acknowledgement of authors' contributions	D	See Author Contributions
**SAMPLE**		
Description	E	ALMC-2 plasma cell line; primary, bone-marrow-derived plasma cells isolated from diagnostic leftovers of bone marrow aspirates
Volume/mass of sample processed	D	1x10^6 ALMC-2 cells were lysed in 1 ml of TRIzol; primary, bone marrow-derived plasma cells obtained from MACS separation (CD138$^+$ selection) were pelleted and lysed in 1 mL of TRIzol
Microdissection or macrodissection	E	Not applicable
Processing procedure	E	See Materials and methods - "Cell culture" and "Isolation of primary bone marrow-derived plasma cells"
If frozen - how and how quickly?	E	Cells were lysed in 1 mL of TRIzol Reagent and stored at -80°C until further processing
If fixed - with what, how quickly?	E	Not applicable
Sample storage conditions and duration (especially for FFPE samples)	E	Samples were kept at -80°C at most up to two months before RNA isolation
**NUCLEIC ACID EXTRACTION**		
Procedure and/or instrumentation	E	See Materials and methods - "RNA extraction and cDNA synthesis"
Name of kit and details of any modifications	E	Rneasy Mini Kit (Qiagen, catalogue number 74106). After gradient centrifugation, the aqueous phase obtained manually was used as starting point for

		extracting the RNA according to the manufacturer's protocol.
Source of additional reagents used	D	Chloroform (Carlo Erba) and TRIzol (Invitrogen, Thermo Fisher, catalogue number 15596026)
Details of DNase or RNAse treatment	E	-
Contamination assessment (DNA or RNA)	E	no-RT controls were prepared for each sample and were included in all the experiments
Nucleic acid quantification	E	RNAs concentration was determined with NanoDrop Spectrophotometer ND-1000 (Thermo Fisher Scientific)
Instrument and method	E	NanoDrop Spectrophotometer ND-1000 (Thermo Fisher Scientific)
Purity (A260/A280)	D	The absorbance ratio 260/280 of all samples proceed was higher than 1.9
Yield	D	-
RNA integrity method/instrument	E	RNAs quality was determined with agarose gel electrophoresis
RIN/RQI or Cq of 3' and 5' transcripts	E	Not applicable
Electrophoresis traces	D	-
Inhibition testing (Cq dilutions, spike or other)	E	No inhibition was noted concerning the results of PCR after samples dilution (dynamic range - see Supplementary figure 5).
**REVERSE TRANSCRIPTION**		
Complete reaction conditions	E	Reverse transcription was performed using the QuantiTect Reverse Transcription kit (Qiagen), according to the manufacturer's instructions.
Amount of RNA and reaction volume	E	Amount of RNA: 1 µg from ALMC-2 cells for dynamic range; 100 ng both from ALMC-2 cells and primary, bone marrow-derived cells for all other studies. Reaction volume: 12 µL of RNAse-free water, as starting volume for the cDNA synthesis protocol
Priming oligonucleotide (if using GSP) and concentration	E	We used the RT primer mix included in the kit, containing an optimized blend of oligo-dT and random primers dissolved in water.

Reverse transcriptase and concentration	E	We used the Quantiscript Reverse Transcriptase included in the kit, containing an optimized mixture of the QIAGEN products Omniscript Reverse Transcriptase and Sensiscript Reverse Transcriptase, which are recombinant heterodimeric enzymes expressed in E. coli. Also contains RNase inhibitor, a 50 kDa protein that strongly inhibits RNases A, B, and C as well as human placental RNases.
Temperature and time	E	According to the manufacturer's instructions: 42°C for 2 min, 42°C for 15 min, 95°C for 3 min
Manufacturer of reagents and catalogue numbers	D	QuantiTect Reverse Transcription kit (Qiagen, catalogue number 205311)
Cqs with and without RT	D	-
Storage conditions of cDNA	D	-20°C
**qPCR TARGET INFORMATION**		
If multiplex, efficiency and LOD of each assay.	E	Not applicable
Sequence accession number	E	See supplementary Table 1
Location of amplicon	D	See supplementary Table 1
Amplicon length	E	See supplementary Table 2
In silico specificity screen (BLAST, etc)	E	See Materials and methods - "Primer design"
Pseudogenes, retropseudogenes or other homologs?	D	See supplementary Table 1
Sequence alignment	D	See supplementary Table 1
Secondary structure analysis of amplicon	D	-
Location of each primer by exon or intron (if applicable)	E	*ACTR3* (forward, f: 11th exon; reverse, r: 11th -12th exon); *ALG9* (f: 10th; r: 10th -11th); AR (f: 1st -2nd; r: 2nd); *CLTC* (f: 2nd; r: 3rd); *ESD* (f: 4th; r: 5th); *HDDC2* (f: 1st; r: 2nd); *HSPA5* (f: 5th -6th; r: 6th); *POLR2B* (f: 2nd; r: 3rd); *SDHA* (f: 12th; r: 12th -13th); *UBB* (f: 1st; r: 1st-2nd); *ACTB* (f: 3rd -4th; r: 4th); *B2M* (f: 1st -2nd; r: 2nd); *GAPDH*

		(f: 3rd -4th; r: 4th); *HMBS* (f: 11th -12th; r: 13th); *HPRT1* (f: 1st -2nd; r: 2nd).
What splice variants are targeted?	E	-
**qPCR OLIGONUCLEOTIDES**		
Primer sequences	E	See supplementary Table 2
RTPrimerDB Identification Number	D	-
Probe sequences	D	Not applicable
Location and identity of any modifications	E	Primer pairs designed for this study have no modifications
Manufacturer of oligonucleotides	D	IDT (Integrated DNA Technologies, Skokie, IL, USA)
Purification method	D	Standard desalting
**qPCR PROTOCOL**		
Complete reaction conditions	E	Every RT-qPCR was realized in triplicates and included no-template controls as well as no-RT control for each gene.
Reaction volume and amount of cDNA/DNA	E	Reaction volume 14 µL; amount of cDNA: 5 µL of cDNA
Primer, (probe), Mg++ and dNTP concentrations	E	Final primer concentration: 0,5 µM; other components are included in the mix (SsoFast EvaGreen Supermix, Biorad) in an unknown concentration
Polymerase identity and concentration	E	Sso7d fusion polymerase
Buffer/kit identity and manufacturer	E	SsoFast EvaGreen Supermix (Biorad, catalogue number 1725202)
Exact chemical constitution of the buffer	D	-
Additives (SYBR Green I, DMSO, etc.)	E	EvaGreen dye
Manufacturer of plates/tubes and catalog number	D	BIO-RAD, product code HSP9655, and MSB1001
Complete thermocycling parameters	E	95°C for 10 min, then 40 cycles of 95°C for 15 s, 60°C for 1 min.

Reaction setup (manual/robotic)	D	Manual
Manufacturer of qPCR instrument	E	C1000 Thermal Cycler CFX96 Real-Time system (Biorad)
**qPCR VALIDATION**		
Evidence of optimization (from gradients)	D	-
Specificity (gel, sequence, melt, or digest)	E	Melting curve analysis, using the following conditions: 95°C for 10 s, 60°C to 95°C for 5 s. For all steps, a ramp rate of 0.5°C/s was used. Agarose gel electrophoresis was performed to verify the unicity and size of each RT-qPCR amplicon.
For SYBR Green I, Cq of the NTC	E	For all the RT-qPCR assays, NTC and no-RT controls showed a Cq > 40 cycles (not detectable)
Standard curves with slope and y-intercept	E	*ACTR3* (Y = -3,440*X + 40,52); *ALG9* (Y = -3,310*X + 41,59); *ESD* (Y = -3,420*X + 41,26); *HDDC2* (Y = -3,452*X + 43,22); *HSPA5* (Y = -3,470*X + 38,51); *POLR2B* (Y = -3,130*X + 41,91); *SDHA* (Y = -3,550*X + 41,03); *ACTB* (Y = -3,277*X + 40,22); *B2M* (Y = -3,429*X + 36,70); *GAPDH* (Y = -3,382*X + 39,19); *HMBS* (Y = -3,389*X + 42,15); *HPRT1* (Y = -3,111*X + 41,09).
PCR efficiency calculated from slope	E	See Supplementary Figure 5
Confidence interval for PCR efficiency or standard error	D	-
r2 of standard curve	E	*ACTR3* (0,9988); *ALG9* (0,9963); *ESD* (0,9978); *HDDC2* (0,9943); *HSPA5* (0,9953); *POLR2B* (0,9889); *SDHA* (0,9945); *ACTB* (0,9940); *B2M* (0,9929); *GAPDH* (0,9950); *HMBS* (0,9945); *HPRT1* (0,9901).
Linear dynamic range	E	Serial dilutions of cDNA over five orders of magnitudes (from 10^{-1} to 10^{-5}) was performed to explore the dynamic range of each assay and calculate PCR efficiency. See Reesults.
Cq variation at lower limit	E	Not detected
Confidence intervals throughout range	D	-

Evidence for limit of detection	E	Not detected, the dilutions of cDNA performed in linear range defined by standard curve
If multiplex, efficiency and LOD of each assay.	E	-
**DATA ANALYSIS**		
qPCR analysis program (source, version)	E	BioRad CFX Manager, version 3.1
Cq method determination	E	Cq determination was set up according to default parameters
Outlier identification and disposition	E	Not applicable
Results of NTCs	E	NTC showed a Cq > 40 cycles (not detectable)
Justification of number and choice of reference genes	E	Not applicable
Description of normalisation method	E	The identification of the most stable gene(s) was performed using RefFinder algorithm, a web-based comprehensive tool integrating the currently available major computational programs (geNorm, NormFinder, BestKeeper, and the comparative Δ-C_t method)
Number and concordance of biological replicates	D	Each sample was run in triplicate, except for experiments assessing intra-assay variation, where 6 technical replicates were performed.
Number and stage (RT or qPCR) of technical replicates	E	For each sample, a single cDNA was prepared, diluted 1:100 or 1:1000, and run in triplicates
Repeatability (intra-assay variation)	E	See Figure 2
Reproducibility (inter-assay variation, %CV)	D	See Figure 2
Power analysis	D	Not applicable
Statistical methods for result significance	E	Not applicable
Software (source, version)	E	GraphPad Prism 8.4.2
Cq or raw data submission using RDML	D	Not applicable

Supplementary Data

List of the 105 papers resulted from the systematic research of published literature on the analysis of candidate reference genes for the normalization of qPCR data in the context of human studies.

1. Abasolo, N.; Torrell, H.; Roig, B.; Moyano, S.; Vilella, E.; Martorell, L., RT-qPCR study on post-mortem brain samples from patients with major psychiatric disorders: reference genes and specimen characteristics. J Psychiatr Res 2011, 45, (11), 1411-8.
2. Adeola, F., Normalization of Gene Expression by Quantitative RT-PCR in Human Cell Line: comparison of 12 Endogenous Reference Genes. Ethiop J Health Sci 2018, 28, (6), 741-748.
3. Andersen, C. L.; Jensen, J. L.; Orntoft, T. F., Normalization of real-time quantitative reverse transcription-PCR data: a model-based variance estimation approach to identify genes suited for normalization, applied to bladder and colon cancer data sets. Cancer Res 2004, 64, (15), 5245-50.
4. Antonov, J.; Goldstein, D. R.; Oberli, A.; Baltzer, A.; Pirotta, M.; Fleischmann, A.; Altermatt, H. J.; Jaggi, R., Reliable gene expression measurements from degraded RNA by quantitative real-time PCR depend on short amplicons and a proper normalization. Lab Invest 2005, 85, (8), 1040-50.
5. Asp, J.; Brantsing, C.; Lovstedt, K.; Benassi, M. S.; Inerot, S.; Gamberi, G.; Picci, P.; Lindahl, A., Evaluation of p16 and Id1 status and endogenous reference genes in human chondrosarcoma by real-time PCR. Int J Oncol 2005, 27, (6), 1577-82.
6. Balogh, A.; Paragh, G., Jr.; Juhasz, A.; Kobling, T.; Torocsik, D.; Miko, E.; Varga, V.; Emri, G.; Horkay, I.; Scholtz, B.; Remenyik, E., Reference genes for quantitative real time PCR in UVB irradiated keratinocytes. J Photochem Photobiol B 2008, 93, (3), 133-9.
7. Borkowska, P.; Zielinska, A.; Paul-Samojedny, M.; Stojko, R.; Kowalski, J., Evaluation of reference genes for quantitative real-time PCR in Wharton's Jelly-derived mesenchymal stem cells after lentiviral transduction and differentiation. Mol Biol Rep 2020, 47, (2), 1107-1115.

8. Brzeszczynska, J.; Brzeszczynski, F.; Samuel, K.; Morgan, K.; Morley, S. D.; Plevris, J. N.; Hayes, P. C., Validation of Reference Genes for Gene Expression Studies by RT-qPCR in HepaRG Cells during Toxicity Testing and Disease Modelling. Cells 2020, 9, (3).
9. Cai, J.; Li, T.; Huang, B.; Cheng, H.; Ding, H.; Dong, W.; Xiao, M.; Liu, L.; Wang, Z., The use of laser microdissection in the identification of suitable reference genes for normalization of quantitative real-time PCR in human FFPE epithelial ovarian tissue samples. PLoS One 2014, 9, (4), e95974.
10. Ceelen, L.; De Craene, J.; De Spiegelaere, W., Evaluation of normalization strategies used in real-time quantitative PCR experiments in HepaRG cell line studies. Clin Chem 2014, 60, (3), 451-4.
11. Chantawibul, S.; Anuwong, A.; Leelawat, K., Validation of appropriate reference genes for gene expression studies in human thyroid gland using real-time RT-PCR. J Med Assoc Thai 2012, 95 Suppl 3, S36-40.
12. Chen, G.; Zhao, L.; Feng, J.; You, G.; Sun, Q.; Li, P.; Han, D.; Zhou, H., Validation of reliable reference genes for real-time PCR in human umbilical vein endothelial cells on substrates with different stiffness. PLoS One 2013, 8, (6), e67360.
13. Chen, J.; Bao, Z.; Huang, Y.; Wang, Z.; Zhao, Y., Selection of Suitable Reference Genes for qPCR Gene Expression Analysis of HepG2 and L02 in Four Different Liver Cell Injured Models. Biomed Res Int 2020, 2020, 8926120.
14. Chey, S.; Claus, C.; Liebert, U. G., Validation and application of normalization factors for gene expression studies in rubella virus-infected cell lines with quantitative real-time PCR. J Cell Biochem 2010, 110, (1), 118-28.
15. Cicinnati, V. R.; Shen, Q.; Sotiropoulos, G. C.; Radtke, A.; Gerken, G.; Beckebaum, S., Validation of putative reference genes for gene expression studies in human hepatocellular carcinoma using real-time quantitative RT-PCR. BMC Cancer 2008, 8, 350.
16. Coulson, D. T.; Brockbank, S.; Quinn, J. G.; Murphy, S.; Ravid, R.; Irvine, G. B.; Johnston, J. A., Identification of valid reference genes for the normalization of RT qPCR gene expression data in human brain tissue. BMC Mol Biol 2008, 9, 46.

17. Curis, E.; Nepost, C.; Grillault Laroche, D.; Courtin, C.; Laplanche, J. L.; Etain, B.; Marie-Claire, C., Selecting reference genes in RT-qPCR based on equivalence tests: a network based approach. Sci Rep 2019, 9, (1), 16231.

18. Curtis, K. M.; Gomez, L. A.; Rios, C.; Garbayo, E.; Raval, A. P.; Perez-Pinzon, M. A.; Schiller, P. C., EF1alpha and RPL13a represent normalization genes suitable for RT-qPCR analysis of bone marrow derived mesenchymal stem cells. BMC Mol Biol 2010, 11, 61.

19. Dang, W.; Zhang, X.; Ma, Q.; Chen, L.; Cao, M.; Miao, J.; Cui, Y.; Zhang, X., Selection of reference genes suitable for normalization of RT-qPCR data in glioma stem cells. Biotechniques 2020, 68, (3), 130-137.

20. de Lazaro, I.; Kostarelos, K., Exposure to graphene oxide sheets alters the expression of reference genes used for real-time RT-qPCR normalization. Sci Rep 2019, 9, (1), 12520.

21. de Lima, C. A. D.; de Lima, S. C.; Barbosa, A. D.; Sandrin-Garcia, P.; de Barros Pita, W.; de Azevedo Silva, J.; Crovella, S., Postmenopausal Osteoporosis reference genes for qPCR expression assays. Sci Rep 2019, 9, (1), 16533.

22. Dean, B.; Udawela, M.; Scarr, E., Validating reference genes using minimally transformed qpcr data: findings in human cortex and outcomes in schizophrenia. BMC Psychiatry 2016, 16, 154.

23. Del Pozo, T.; Gutierrez-Garcia, R.; Latorre, M.; Gonzalez, M.; Suazo, M., Identification of reference genes for quantitative real-time PCR studies in human cell lines under copper and zinc exposure. Biometals 2016, 29, (5), 935-44.

24. El-Kashef, N.; Gomes, I.; Mercer-Chalmers-Bender, K.; Schneider, P. M.; Rothschild, M. A.; Juebner, M., Validation of adequate endogenous reference genes for reverse transcription-qPCR studies in human post-mortem brain tissue of SIDS cases. Forensic Sci Med Pathol 2015, 11, (4), 517-29.

25. Erickson, H. S.; Albert, P. S.; Gillespie, J. W.; Wallis, B. S.; Rodriguez-Canales, J.; Linehan, W. M.; Gonzalez, S.; Velasco, A.; Chuaqui, R. F.; Emmert-Buck, M. R., Assessment of normalization strategies for quantitative RT-PCR using microdissected tissue samples. Lab Invest 2007, 87, (9), 951-62.

26. Fassunke, J.; Blum, M. C.; Schildhaus, H. U.; Zapatka, M.; Brors, B.; Kunstlinger, H.; Buttner, R.; Wardelmann, E.; Merkelbach-Bruse, S., qPCR in gastrointestinal stromal tumors: Evaluation of reference genes and expression analysis of KIT and the alternative receptor tyrosine kinases FLT3, CSF1-R, PDGFRB, MET and AXL. BMC Mol Biol 2010, 11, 100.

27. Ferreira, E.; Cronje, M. J., Selection of suitable reference genes for quantitative real-time PCR in apoptosis-induced MCF-7 breast cancer cells. Mol Biotechnol 2012, 50, (2), 121-8.

28. Fjeldbo, C. S.; Aarnes, E. K.; Malinen, E.; Kristensen, G. B.; Lyng, H., Identification and Validation of Reference Genes for RT-qPCR Studies of Hypoxia in Squamous Cervical Cancer Patients. PLoS One 2016, 11, (5), e0156259.

29. Fu, J.; Bian, L.; Zhao, L.; Dong, Z.; Gao, X.; Luan, H.; Sun, Y.; Song, H., Identification of genes for normalization of quantitative real-time PCR data in ovarian tissues. Acta Biochim Biophys Sin (Shanghai) 2010, 42, (8), 568-74.

30. Fu, L. Y.; Jia, H. L.; Dong, Q. Z.; Wu, J. C.; Zhao, Y.; Zhou, H. J.; Ren, N.; Ye, Q. H.; Qin, L. X., Suitable reference genes for real-time PCR in human HBV-related hepatocellular carcinoma with different clinical prognoses. BMC Cancer 2009, 9, 49.

31. Gao, Q.; Wang, X. Y.; Fan, J.; Qiu, S. J.; Zhou, J.; Shi, Y. H.; Xiao, Y. S.; Xu, Y.; Huang, X. W.; Sun, J., Selection of reference genes for real-time PCR in human hepatocellular carcinoma tissues. J Cancer Res Clin Oncol 2008, 134, (9), 979-86.

32. Gentile, A. M.; Lhamyani, S.; Coin-Araguez, L.; Oliva-Olivera, W.; Zayed, H.; Vega-Rioja, A.; Monteseirin, J.; Romero-Zerbo, S. Y.; Tinahones, F. J.; Bermudez-Silva, F. J.; El Bekay, R., RPL13A and EEF1A1 Are Suitable Reference Genes for qPCR during Adipocyte Differentiation of Vascular Stromal Cells from Patients with Different BMI and HOMA-IR. PLoS One 2016, 11, (6), e0157002.

33. Giricz, O.; Lauer-Fields, J. L.; Fields, G. B., The normalization of gene expression data in melanoma: investigating the use of glyceraldehyde 3-phosphate dehydrogenase and 18S ribosomal RNA as internal reference genes for quantitative real-time PCR. Anal Biochem 2008, 380, (1), 137-9.

34. Gonzalez-Bermudez, L.; Anglada, T.; Genesca, A.; Martin, M.; Terradas, M., Identification of reference genes for RT-qPCR data normalisation in aging studies. Sci Rep 2019, 9, (1), 13970.

35. Green, T. M.; de Stricker, K.; Moller, M. B., Validation of putative reference genes for normalization of Q-RT-PCR data from paraffin-embedded lymphoid tissue. Diagn Mol Pathol 2009, 18, (4), 243-9.
36. Grube, S.; Gottig, T.; Freitag, D.; Ewald, C.; Kalff, R.; Walter, J., Selection of suitable reference genes for expression analysis in human glioma using RT-qPCR. J Neurooncol 2015, 123, (1), 35-42.
37. Hamalainen, H. K.; Tubman, J. C.; Vikman, S.; Kyrola, T.; Ylikoski, E.; Warrington, J. A.; Lahesmaa, R., Identification and validation of endogenous reference genes for expression profiling of T helper cell differentiation by quantitative real-time RT-PCR. Anal Biochem 2001, 299, (1), 63-70.
38. Hampton, T. H.; Koeppen, K.; Bashor, L.; Stanton, B. A., Selection of reference genes for quantitative PCR: identifying reference genes for airway epithelial cells exposed to Pseudomonas aeruginosa. Am J Physiol Lung Cell Mol Physiol 2020, 319, (2), L256-L265.
39. Henn, D.; Bandner-Risch, D.; Perttunen, H.; Schmied, W.; Porras, C.; Ceballos, F.; Rodriguez-Losada, N.; Schafers, H. J., Identification of reference genes for quantitative RT-PCR in ascending aortic aneurysms. PLoS One 2013, 8, (1), e54132.
40. Jacob, F.; Guertler, R.; Naim, S.; Nixdorf, S.; Fedier, A.; Hacker, N. F.; Heinzelmann-Schwarz, V., Careful selection of reference genes is required for reliable performance of RT-qPCR in human normal and cancer cell lines. PLoS One 2013, 8, (3), e59180.
41. Jain, N.; Nitisa, D.; Pirsko, V.; Cakstina, I., Selecting suitable reference genes for qPCR normalization: a comprehensive analysis in MCF-7 breast cancer cell line. BMC Mol Cell Biol 2020, 21, (1), 68.
42. Janik, M. E.; Szwed, S.; Grzmil, P.; Kaczmarek, R.; Czerwinski, M.; Hoja-Lukowicz, D., RT-qPCR analysis of human melanoma progression-related genes - A novel workflow for selection and validation of candidate reference genes. Int J Biochem Cell Biol 2018, 101, 12-18.
43. Javadirad, S. M.; Mokhtari, M.; Esfandiarpour, G.; Kolahdouzan, M., The pseudogene problem and RT-qPCR data normalization; SYMPK: a suitable reference gene for papillary thyroid carcinoma. Sci Rep 2020, 10, (1), 18408.

44. Jung, M.; Ramankulov, A.; Roigas, J.; Johannsen, M.; Ringsdorf, M.; Kristiansen, G.; Jung, K., In search of suitable reference genes for gene expression studies of human renal cell carcinoma by real-time PCR. BMC Mol Biol 2007, 8, 47.

45. Kaszubowska, L.; Wierzbicki, P. M.; Karsznia, S.; Damska, M.; Slebioda, T. J.; Foerster, J.; Kmiec, Z., Optimal reference genes for qPCR in resting and activated human NK cells--Flow cytometric data correspond to qPCR gene expression analysis. J Immunol Methods 2015, 422, 125-9.

46. Khanna, P.; Johnson, K. L.; Maron, J. L., Optimal reference genes for RT-qPCR normalization in the newborn. Biotech Histochem 2017, 92, (7), 459-466.

47. Kidd, M.; Nadler, B.; Mane, S.; Eick, G.; Malfertheiner, M.; Champaneria, M.; Pfragner, R.; Modlin, I., GeneChip, geNorm, and gastrointestinal tumors: novel reference genes for real-time PCR. Physiol Genomics 2007, 30, (3), 363-70.

48. Kilic, Y.; Celebiler, A. C.; Sakizli, M., Selecting housekeeping genes as references for the normalization of quantitative PCR data in breast cancer. Clin Transl Oncol 2014, 16, (2), 184-90.

49. Kirschneck, C.; Batschkus, S.; Proff, P.; Kostler, J.; Spanier, G.; Schroder, A., Valid gene expression normalization by RT-qPCR in studies on hPDL fibroblasts with focus on orthodontic tooth movement and periodontitis. Sci Rep 2017, 7, (1), 14751.

50. Koppelkamm, A.; Vennemann, B.; Fracasso, T.; Lutz-Bonengel, S.; Schmidt, U.; Heinrich, M., Validation of adequate endogenous reference genes for the normalisation of qPCR gene expression data in human post mortem tissue. Int J Legal Med 2010, 124, (5), 371-80.

51. Kozmus, C. E.; Potocnik, U., Reference genes for real-time qPCR in leukocytes from asthmatic patients before and after anti-asthma treatment. Gene 2015, 570, (1), 71-7.

52. Leal, M. F.; Arliani, G. G.; Astur, D. C.; Franciozi, C. E.; Debieux, P.; Andreoli, C. V.; Smith, M. C.; Pochini, A. C.; Ejnisman, B.; Cohen, M., Comprehensive selection of reference genes for expression studies in meniscus injury using quantitative real-time PCR. Gene 2016, 584, (1), 60-68.

53. Leal, M. F.; Astur, D. C.; Debieux, P.; Arliani, G. G.; Silveira Franciozi, C. E.; Loyola, L. C.; Andreoli, C. V.; Smith, M. C.; Pochini Ade, C.; Ejnisman, B.; Cohen, M., Identification of Suitable

Reference Genes for Investigating Gene Expression in Anterior Cruciate Ligament Injury by Using Reverse Transcription-Quantitative PCR. PLoS One 2015, 10, (7), e0133323.
54. Leduc, V.; Legault, V.; Dea, D.; Poirier, J., Normalization of gene expression using SYBR green qPCR: a case for paraoxonase 1 and 2 in Alzheimer's disease brains. J Neurosci Methods 2011, 200, (1), 14-9.
55. Leitao Mda, C.; Coimbra, E. C.; de Lima Rde, C.; Guimaraes Mde, L.; Heraclio Sde, A.; Silva Neto Jda, C.; de Freitas, A. C., Quantifying mRNA and microRNA with qPCR in cervical carcinogenesis: a validation of reference genes to ensure accurate data. PLoS One 2014, 9, (11), e111021.
56. Li, L.; Yan, Y.; Xu, H.; Qu, T.; Wang, B., Selection of reference genes for gene expression studies in ultraviolet B-irradiated human skin fibroblasts using quantitative real-time PCR. BMC Mol Biol 2011, 12, 8.
57. Li, T.; Diao, H.; Zhao, L.; Xing, Y.; Zhang, J.; Liu, N.; Yan, Y.; Tian, X.; Sun, W.; Liu, B., Identification of suitable reference genes for real-time quantitative PCR analysis of hydrogen peroxide-treated human umbilical vein endothelial cells. BMC Mol Biol 2017, 18, (1), 10.
58. Li, X.; Yang, Q.; Bai, J.; Xuan, Y.; Wang, Y., Identification of appropriate reference genes for human mesenchymal stem cell analysis by quantitative real-time PCR. Biotechnol Lett 2015, 37, (1), 67-73.
59. Li, X.; Yang, Q.; Bai, J.; Yang, Y.; Zhong, L.; Wang, Y., Identification of optimal reference genes for quantitative PCR studies on human mesenchymal stem cells. Mol Med Rep 2015, 11, (2), 1304-11.
60. Li, Y.; Lu, H.; Ji, Y.; Wu, S.; Yang, Y., Identification of genes for normalization of real-time RT-PCR data in placental tissues from intrahepatic cholestasis of pregnancy. Placenta 2016, 48, 133-135.
61. Liu, L. L.; Zhao, H.; Ma, T. F.; Ge, F.; Chen, C. S.; Zhang, Y. P., Identification of valid reference genes for the normalization of RT-qPCR expression studies in human breast cancer cell lines treated with and without transient transfection. PLoS One 2015, 10, (1), e0117058.

62. Liu, S.; Zhu, P.; Zhang, L.; Ding, S.; Zheng, S.; Wang, Y.; Lu, F., Selection of reference genes for RT-qPCR analysis in tumor tissues from male hepatocellular carcinoma patients with hepatitis B infection and cirrhosis. Cancer Biomark 2013, 13, (5), 345-9.
63. Liu, X.; Xie, J.; Liu, Z.; Gong, Q.; Tian, R.; Su, G., Identification and validation of reference genes for quantitative RT-PCR analysis of retinal pigment epithelium cells under hypoxia and/or hyperglycemia. Gene 2016, 580, (1), 41-6.
64. Lyng, M. B.; Laenkholm, A. V.; Pallisgaard, N.; Ditzel, H. J., Identification of genes for normalization of real-time RT-PCR data in breast carcinomas. BMC Cancer 2008, 8, 20.
65. Moermans, C.; Deliege, E.; Pirottin, D.; Poulet, C.; Guiot, J.; Henket, M.; da Silva, J.; Louis, R., Suitable reference genes determination for real-time PCR using induced sputum samples. Eur Respir J 2019, 54, (6).
66. Mohelnikova-Duchonova, B.; Oliverius, M.; Honsova, E.; Soucek, P., Evaluation of reference genes and normalization strategy for quantitative real-time PCR in human pancreatic carcinoma. Dis Markers 2012, 32, (3), 203-10.
67. Mori, R.; Wang, Q.; Danenberg, K. D.; Pinski, J. K.; Danenberg, P. V., Both beta-actin and GAPDH are useful reference genes for normalization of quantitative RT-PCR in human FFPE tissue samples of prostate cancer. Prostate 2008, 68, (14), 1555-60.
68. Mossberg, K.; Svensson, P. A.; Gidlof, O.; Erlinge, D.; Jern, S.; Brogren, H., Normalization of qPCR in platelets - YWHAE a potential genericreference gene. Platelets 2016, 27, (8), 729-734.
69. Nakayama, T.; Okada, N.; Yoshikawa, M.; Asaka, D.; Kuboki, A.; Kojima, H.; Tanaka, Y.; Haruna, S. I., Assessment of suitable reference genes for RT-qPCR studies in chronic rhinosinusitis. Sci Rep 2018, 8, (1), 1568.
70. Neville, M. J.; Collins, J. M.; Gloyn, A. L.; McCarthy, M. I.; Karpe, F., Comprehensive human adipose tissue mRNA and microRNA endogenous control selection for quantitative real-time-PCR normalization. Obesity (Silver Spring) 2011, 19, (4), 888-92.
71. Nielsen, S.; Bassler, N.; Grzanka, L.; Swakon, J.; Olko, P.; Andreassen, C. N.; Alsner, J.; Sorensen, B. S., Optimal reference genes for normalization of qPCR gene expression data from proton and photon irradiated dermal fibroblasts. Sci Rep 2018, 8, (1), 12688.

72. Normann, K. R.; Oystese, K. A. B.; Berg, J. P.; Lekva, T.; Berg-Johnsen, J.; Bollerslev, J.; Olarescu, N. C., Selection and validation of reliable reference genes for RT-qPCR analysis in a large cohort of pituitary adenomas. Mol Cell Endocrinol 2016, 437, 183-189.

73. Okamura, K.; Inagaki, Y.; Matsui, T. K.; Matsubayashi, M.; Komeda, T.; Ogawa, M.; Mori, E.; Tanaka, Y., RT-qPCR analyses on the osteogenic differentiation from human iPS cells: an investigation of reference genes. Sci Rep 2020, 10, (1), 11748.

74. O'Shaughnessy, P. J.; Monteiro, A.; Fowler, P. A., Identification of stable endogenous reference genes for real-time PCR in the human fetal gonad using an external standard technique. Mol Hum Reprod 2011, 17, (10), 620-5.

75. Palombella, S.; Pirrone, C.; Cherubino, M.; Valdatta, L.; Bernardini, G.; Gornati, R., Identification of reference genes for qPCR analysis during hASC long culture maintenance. PLoS One 2017, 12, (2), e0170918.

76. Piana, C.; Wirth, M.; Gerbes, S.; Viernstein, H.; Gabor, F.; Toegel, S., Validation of reference genes for qPCR studies on Caco-2 cell differentiation. Eur J Pharm Biopharm 2008, 69, (3), 1187-92.

77. Rho, H. W.; Lee, B. C.; Choi, E. S.; Choi, I. J.; Lee, Y. S.; Goh, S. H., Identification of valid reference genes for gene expression studies of human stomach cancer by reverse transcription-qPCR. BMC Cancer 2010, 10, 240.

78. Ribeiro, M. A.; dos Reis, M. B.; de Moraes, L. N.; Briton-Jones, C.; Rainho, C. A.; Scarano, W. R., Defining suitable reference genes for RT-qPCR analysis on human sertoli cells after 2,3,7,8-tetrachlorodibenzo-p-dioxin (TCDD) exposure. Mol Biol Rep 2014, 41, (11), 7063-6.

79. Riedel, G.; Rudrich, U.; Fekete-Drimusz, N.; Manns, M. P.; Vondran, F. W.; Bock, M., An extended DeltaCT-method facilitating normalisation with multiple reference genes suited for quantitative RT-PCR analyses of human hepatocyte-like cells. PLoS One 2014, 9, (3), e93031.

80. Rohn, G.; Koch, A.; Krischek, B.; Stavrinou, P.; Goldbrunner, R.; Timmer, M., ACTB and SDHA Are Suitable Endogenous Reference Genes for Gene Expression Studies in Human Astrocytomas Using Quantitative RT-PCR. Technol Cancer Res Treat 2018, 17, 1533033818802318.

81. Royer, C.; Begin, A. G.; Plawinski, L.; Levesque, L.; Durrieu, M. C.; Laroche, G., Validation of reference genes for real-time PCR of cord blood mononuclear cells, differentiating endothelial progenitor cells, and mature endothelial cells. Exp Cell Res 2018, 370, (2), 389-398.

82. Rueda-Martinez, C.; Lamas, O.; Mataro, M. J.; Robledo-Carmona, J.; Sanchez-Espin, G.; Jimenez-Navarro, M.; Such-Martinez, M.; Fernandez, B., Selection of reference genes for quantitative real time PCR (qPCR) assays in tissue from human ascending aorta. PLoS One 2014, 9, (5), e97449.

83. Rydbirk, R.; Folke, J.; Winge, K.; Aznar, S.; Pakkenberg, B.; Brudek, T., Assessment of brain reference genes for RT-qPCR studies in neurodegenerative diseases. Sci Rep 2016, 6, 37116.

84. Silberberg, G.; Baruch, K.; Navon, R., Detection of stable reference genes for real-time PCR analysis in schizophrenia and bipolar disorder. Anal Biochem 2009, 391, (2), 91-7.

85. Soes, S.; Sorensen, B. S.; Alsner, J.; Overgaard, J.; Hager, H.; Hansen, L. L.; Kristensen, L. S., Identification of accurate reference genes for RT-qPCR analysis of formalin-fixed paraffin-embedded tissue from primary non-small cell lung cancers and brain and lymph node metastases. Lung Cancer 2013, 81, (2), 180-6.

86. Song, W.; Zhang, W. H.; Zhang, H.; Li, Y.; Zhang, Y.; Yin, W.; Yang, Q., Validation of housekeeping genes for the normalization of RT-qPCR expression studies in oral squamous cell carcinoma cell line treated by 5 kinds of chemotherapy drugs. Cell Mol Biol (Noisy-le-grand) 2016, 62, (13), 29-34.

87. Tan, S. C.; Ismail, M. P.; Duski, D. R.; Othman, N. H.; Bhavaraju, V. M.; Ankathil, R., Identification of Optimal Reference Genes for Normalization of RT-qPCR Data in Cancerous and Non-Cancerous Tissues of Human Uterine Cervix. Cancer Invest 2017, 35, (3), 163-173.

88. Toegel, S.; Huang, W.; Piana, C.; Unger, F. M.; Wirth, M.; Goldring, M. B.; Gabor, F.; Viernstein, H., Selection of reliable reference genes for qPCR studies on chondroprotective action. BMC Mol Biol 2007, 8, 13.

89. Usarek, E.; Baranczyk-Kuzma, A.; Kazmierczak, B.; Gajewska, B.; Kuzma-Kozakiewicz, M., Validation of qPCR reference genes in lymphocytes from patients with amyotrophic lateral sclerosis. PLoS One 2017, 12, (3), e0174317.

90. Vandesompele, J.; De Preter, K.; Pattyn, F.; Poppe, B.; Van Roy, N.; De Paepe, A.; Speleman, F., Accurate normalization of real-time quantitative RT-PCR data by geometric averaging of multiple internal control genes. Genome Biol 2002, 3, (7), RESEARCH0034.

91. Vreeburg, R. A.; Bastiaan-Net, S.; Mes, J. J., Normalization genes for quantitative RT-PCR in differentiated Caco-2 cells used for food exposure studies. Food Funct 2011, 2, (2), 124-9.

92. Wang, H.; Yang, B.; Geng, T.; Li, B.; Dai, P.; Chen, C., Tissue-specific selection of optimal reference genes for expression analysis of anti-cancer drug-related genes in tumor samples using quantitative real-time RT-PCR. Exp Mol Pathol 2015, 98, (3), 375-81.

93. Wang, Q.; Ishikawa, T.; Michiue, T.; Zhu, B. L.; Guan, D. W.; Maeda, H., Stability of endogenous reference genes in postmortem human brains for normalization of quantitative real-time PCR data: comprehensive evaluation using geNorm, NormFinder, and BestKeeper. Int J Legal Med 2012, 126, (6), 943-52.

94. Weber, R.; Bertoni, A. P.; Bessestil, L. W.; Brasil, B. M.; Brum, L. S.; Furlanetto, T. W., Validation of reference genes for normalization gene expression in reverse transcription quantitative PCR in human normal thyroid and goiter tissue. Biomed Res Int 2014, 2014, 198582.

95. Weiss, J.; Theile, D.; Haefeli, W. E., Rifampicin alters the expression of reference genes used to normalize real-time quantitative RT-PCR data. Naunyn Schmiedebergs Arch Pharmacol 2012, 385, (10), 1025-34.

96. Wisnieski, F.; Calcagno, D. Q.; Leal, M. F.; dos Santos, L. C.; Gigek Cde, O.; Chen, E. S.; Pontes, T. B.; Assumpcao, P. P.; de Assumpcao, M. B.; Demachki, S.; Burbano, R. R.; Smith Mde, A., Reference genes for quantitative RT-PCR data in gastric tissues and cell lines. World J Gastroenterol 2013, 19, (41), 7121-8.

97. Xie, J.; Liu, X.; Li, Y.; Liu, Y.; Su, G., Validation of RT-qPCR reference genes and determination of Robo4 expression levels in human retinal endothelial cells under hypoxia and/or hyperglycemia. Gene 2016, 585, (1), 135-142.

98. Yang, X.; Hatfield, J. T.; Hinze, S. J.; Mu, X.; Anderson, P. J.; Powell, B. C., Bone to pick: the importance of evaluating reference genes for RT-qPCR quantification of gene expression in craniosynostosis and bone-related tissues and cells. BMC Res Notes 2012, 5, 222.

99. Yin, W. Z.; Yang, Q. W.; Niu, K.; Ren, M.; He, D.; Song, W. Z., Validation of reference genes for the normalization of RT-qPCR expression studies on human laryngeal cancer and hypopharyngeal cancer. Eur Rev Med Pharmacol Sci 2019, 23, (10), 4199-4209.

100. Zarybnicky, T.; Matouskova, P.; Ambroz, M.; Subrt, Z.; Skalova, L.; Bousova, I., The Selection and Validation of Reference Genes for mRNA and microRNA Expression Studies in Human Liver Slices Using RT-qPCR. Genes (Basel) 2019, 10, (10).

101. Zhang, H.; Guan, Z. S.; Guan, S. H.; Yang, K.; Pan, Y.; Wu, Y. Y.; Wang, A. H.; Sun, B. B.; Hou, J.; Mu, X. X.; Gao, Y. F.; Cheng, W. S., Identification of Suitable Candidate Reference Genes for Gene Expression Analysis by RT-qPCR in Peripheral Blood Mononuclear Cells of CHB Patients. Clin Lab 2016, 62, (1-2), 227-34.

102. Zhang, X.; Ding, L.; Sandford, A. J., Selection of reference genes for gene expression studies in human neutrophils by real-time PCR. BMC Mol Biol 2005, 6, 4.

103. Zhao, J.; Zhou, H.; Sun, L.; Yang, B.; Zhang, L.; Shi, H.; Zheng, Y., Selection of suitable reference genes for quantitative real-time PCR in trabecular meshwork cells under oxidative stress. Free Radic Res 2017, 51, (1), 103-111.

104. Zhu, X.; Zhang, L.; Hu, Y.; Zhang, J., Identification of suitable reference genes for real-time qPCR in homocysteine-treated human umbilical vein endothelial cells. PLoS One 2018, 13, (12), e0210087.

105. Zyzynska-Granica, B.; Koziak, K., Identification of suitable reference genes for real-time PCR analysis of statin-treated human umbilical vein endothelial cells. PLoS One 2012, 7, (12), e51547.
